# Sustainable Synthesis of Defect-Rich NiO Nanoparticles from Seaweed for Enhanced Oxygen Evolution

**DOI:** 10.1021/acsomega.6c05066

**Published:** 2026-08-24

**Authors:** Adiel Soares Ferreira, Johnnys da Silva Hortêncio, André Luiz Menezes de Oliveira, Rodolfo B. da Silva, Nykon Craveiro, José Souto Rosa-Filho, Fausthon Fred da Silva

**Affiliations:** † Programa de Pós-Graduação em Química, Universidade Federal Rural de Pernambuco (UFRPE), 52171-900 Recife, PE, Brazil; ‡ Programa de Pós-Graduação em Química, Universidade Federal da Paraíba (UFPB), 58.051-900 João Pessoa, PB, Brazil; § Núcleo de Pesquisa e Extensão LACOM, Universidade Federal da Paraíba (UFPB), 58051-900 João Pessoa, PB, Brazil; ∥ Laboratório Institucional de Microscopia e Caracterização de Materiais, Depto. de Engenharia de Materiais, Universidade Federal do Rio Grande do Norte, 59078-970 Natal, RN, Brazil; ⊥ Programa de Pós-Graduação em Ciência e Engenharia de Materiais—PPCEM, Universidade Federal da Paraíba (UFPB), 58.051-900 João Pessoa, PB, Brazil; # Departamento de Física, 89113 Universidade do Estado do Rio Grande do Norte (UERN), 59610-090 Mossoró, RN, Brazil; ∇ Laboratório de Bentos (LaBen), Departamento de Oceanografia, 28116 Universidade Federal de Pernambuco, Av. Prof. Moraes Rêgo, 1235, 50670-901 Recife, PE, Brazil; ○ Departamento de Química, Universidade Federal da Paraíba (UFPB), 58051-900 João Pessoa, PB, Brazil; ◆ Departamento de Química Fundamental, Universidade Federal de Pernambuco (UFPE), 50670-901 Recife, PE, Brazil

## Abstract

Green synthesis has emerged as a sustainable alternative for preparing nanostructured electrocatalysts using renewable biological resources. In this work, NiO nanoparticles were synthesized using an ethanolic extract of *Sargassum sp.* as a biogenic mediator, and the influence of calcination temperature (300, 500, and 700 °C) on their physicochemical and electrocatalytic properties was systematically investigated. X-ray diffraction confirmed the formation of NiO cubic phase and showed an increase in crystallite size with increasing calcination temperature. FTIR, Raman, XPS, and EPR analyses revealed temperature-dependent changes in surface composition and defect chemistry, including mixed Ni^2+^/Ni^3+^ species and oxygen-vacancy-related centers. EPR signals at *g* ≈ 2.0005–2.0006 are assigned to *F* ^+^-type centers. Electrochemical measurements in 1.0 mol L^–1^ KOH showed that NiO-300 exhibited the highest OER activity, requiring an overpotential of 352 mV to reach 10 mA cm^–2^, compared with 368 and 356 mV for NiO-500 and NiO-700, respectively. Although NiO-300 displayed the lowest estimated electrochemically active surface area among the samples, it consistently showed the lowest charge-transfer resistance, indicating more favorable interfacial kinetics. Its enhanced performance is therefore associated with the combined effects of defect-related surface species, oxygen-vacancy-related centers, and improved charge-transfer dynamics. Chronopotentiometric measurements at 10 and 100 mA cm^–2^ further demonstrated the excellent electrochemical stability of NiO-300, while post-OER XRD and Raman analyses confirmed the preservation of its crystalline framework after prolonged operation.

## Graphical abstract

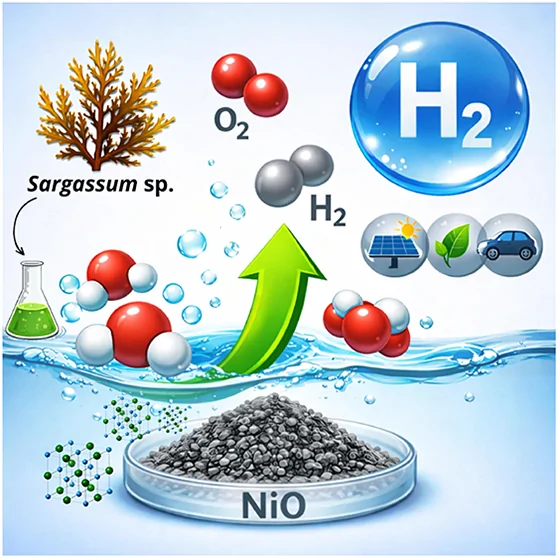

## 1 Introduction

Economic development and population growth have led to a continuous increase in global energy demand, which is still predominantly supplied by fossil fuels such as oil, coal, and natural gas, the main contributors to CO_2_ emissions and global warming.^1,2^ In this context, hydrogen (H_2_) has emerged as a promising clean energy carrier for achieving carbon neutrality and meeting the 2050 energy transition goals.^3^ Although hydrogen can be produced through several pathways, most of the current global production still relies on steam methane reforming, a fossil-based process associated with substantial CO_2_ emissions.^4^ Green hydrogen, in contrast, is produced by water electrolysis powered by renewable electricity sources such as solar energy, generating H_2_ and O_2_ without direct carbon emissions.^5^ However, despite its theoretical cell potential of 1.23 V, water electrolysis requires additional energy because of kinetic limitations, particularly those associated with the oxygen evolution reaction (OER), a complex four-electron oxidation process that represents the main bottleneck for efficient hydrogen production.^6–9^

To overcome this limitation, highly active electrocatalysts are required to reduce the OER overpotential and improve reaction kinetics.^10^ Although RuO_2_- and IrO_2_-based catalysts remain the benchmark materials, their high cost and limited availability hinder large-scale implementation.^11,12^ Consequently, increasing attention has been devoted to earth-abundant transition metal oxides based on Co, Ni, Fe, and Mn.^13–16^ Among them, Ni/NiO-based materials have attracted particular interest owing to their low cost, natural abundance, low toxicity, and excellent catalytic activity and stability under alkaline conditions.^16^ NiO has been synthesized through several conventional methods, including hydrothermal, precipitation, sol–gel, and MOF-derived approaches.^17,18^ More recently, green synthesis strategies employing plant extracts, fungi, bacteria, and seaweed have emerged as sustainable alternatives because biological metabolites can simultaneously act as reducing, complexing, and stabilizing agents, enabling environmentally friendly, low-cost, and potentially scalable synthesis routes compared with conventional physicochemical methods.^19–24^

In recent years, numerous green synthesis strategies have been developed for NiO nanoparticles, employing a wide variety of biological resources as reducing and stabilizing agents.^16,24–26^ Plant extracts remain the most explored systems owing to their high content of flavonoids, phenolics, sugars, and amino acids, whereas microorganisms offer excellent control over nucleation but often require complex cultivation protocols.^25,26^ Algae have recently emerged as attractive alternatives because of their rich polysaccharide and polyphenol composition, rapid biomass production, and minimal cultivation requirements.^23^ Nevertheless, despite increasing interest in algae-mediated synthesis, studies employing marine macroalgae for preparing electrocatalytically active NiO remain very limited, particularly for oxygen evolution applications.

Marine macroalgae are among the oldest multicellular organisms on Earth, with an evolutionary history spanning more than one billion years.^27,28^ Their adaptation to dynamic and often harsh marine environments has promoted extensive metabolic diversification, resulting in a remarkable abundance of primary and secondary metabolites with significant ecological and biotechnological value.^29,30^ Along the tropical Brazilian coast, seaweeds are highly abundant and widely distributed throughout the year, making them an inexpensive and renewable biomass source.^31^ Compared with terrestrial plants, marine macroalgae offer several advantages as green synthetic platforms, including rapid biomass production without the need for arable land or freshwater, high availability, and elevated concentrations of sulfated polysaccharides, alginates, fucoidans, proteins, pigments, and polyphenols, which can simultaneously act as reducing, chelating, and stabilizing agents during nanoparticle synthesis.^32^

Unlike microorganism-mediated synthesis, seaweed-based routes do not require sterile cultivation conditions or prolonged incubation periods, making the process simpler, more economical, and potentially scalable.^23,32,33^ Furthermore, the valorization of abundant *Sargassum* biomass contributes to waste management and supports circular bioeconomy strategies. During the green synthesis process, the diverse metabolites present in seaweed extracts promote the bioreduction of metal ions and nanoparticle formation through three main stages: (i) activation, involving reduction and nucleation; (ii) spontaneous coalescence of primary nanoparticles through heterogeneous nucleation and successive metal-ion reduction, leading to greater thermodynamic stability; and (iii) termination, which determines the final morphology and surface characteristics of the nanoparticles.^34,35^

NiO nanoparticles mediated by aqueous extract of red algae were synthesized with an average size of 32.64 nm, applied as a high-efficiency catalyst for the preparation of pyridopyrimidine derivatives.^36^ Green synthesized NiO using plant extracts for OER exhibited an overpotential of 413 mV at 10 mA cm^–2^ and a Tafel slope of 95 mV dec^–1^.^37^ Zahra et al. also developed a new green NiO:ZnO/PdO nanocomposite with an overpotential of 410 mV and a Tafel slope of 76 mV dec^–1^.^38^ Furthermore, our research group performed the green synthesis of NiO mediated by *Ipomoea asarifolia* extract, applied to OER, with an overpotential of only 307 mV at 10 mA cm^–2^, and a Tafel slope of 60.35 mV dec^–1^.^39^ Although these studies demonstrate the potential of green synthesized NiO, they are almost exclusively based on terrestrial plant extracts, while marine macroalgae remain largely unexplored for OER electrocatalysis.^23,24,33,40^

Despite these advances, important knowledge gaps remain. Most green synthesis strategies reported for NiO rely on terrestrial plant extracts, while marine biomass remains largely unexplored. Furthermore, the influence of calcination temperature on the physicochemical evolution of seaweed-derived NiO and its relationship with electrocatalytic OER performance has not yet been systematically investigated. Among marine macroalgae, *Sargassum* species are particularly attractive because they are among the most abundant brown algae along tropical coastlines and are naturally rich in alginates, fucoidans, polyphenols, proteins, and other oxygenated metabolites that can act as reducing, chelating, and stabilizing agents during metal-ion reduction, thereby promoting nanoparticle nucleation and growth.^29,32^ Thus, this study presents a new synthetic route for obtaining NiO nanoparticles using an ethanolic extract of *Sargassum sp.* biomass and investigates the influence of calcination temperature on their physicochemical properties and OER electrocatalytic performance through comprehensive physicochemical and electrochemical characterization.

## 2 Experimental Section

### 2.1 Materials

Nickel nitrate hexahydrate (Ni­(NO_3_)_2_·6H_2_O, 99%) and ethanol (99%) were supplied by Dinâmica Química. Nafion copolymer, hydrochloric acid (37%), sodium hydroxide (99%), and Isopropyl alcohol (99%) were purchased from Sigma-Aldrich. Nickel foam (Ni 99.8%, porosity >95%) was acquired from QiJing Ltd. (China). All reagents were used without further purification.

### 2.2 Collection and Pretreatment of the Sargassum sp

*Sargassum sp* biomass was collected during the rainy season (March to August) on Enseada dos Corrais beach, located in the municipality of Cabo de Santo Agostinho, Pernambuco, on the northeastern coast of Brazil. The collected *Sargassum* samples were washed with distilled water, air-dried at room temperature, crushed, and stored for later preparation of the ethanolic extract.

### 2.3 Preparation of Ethanolic Extract

The ethanolic extract was produced with 4 g of dried Sargassum in 100 mL of ethanol. The system was maintained at 40 °C for 2 h under continuous mechanical agitation. The residual biomass was filtered off, the resulting solution was transferred to an amber bottle, and stored at 5 °C.

### 2.4 Synthesis of the NiO Nanoparticles

The seaweed extract (3 mL) was diluted with ethanol (27 mL) (1:9, 30 mL total) in a beaker. After this, 1 g of nickel nitrate hexahydrate was added. The solution was heated at 60 °C under magnetic agitation until solvent evaporation. Then, the resulting slurry was transferred to an alumina crucible and calcined at 300 °C for 2 h under air to generate the NiO nanoparticles. This sample was named NiO-300. Different samples were obtained by varying the calcination temperature to 500 °C (NiO-500) and 700 °C (NiO-700). Figure 1 shows the schematic representation of the experimental procedure. For comparative purposes, a sample was prepared using only water, following the same experimental procedure, followed by calcination at 300 °C (named NiO­(w)).

**Figure 1:**
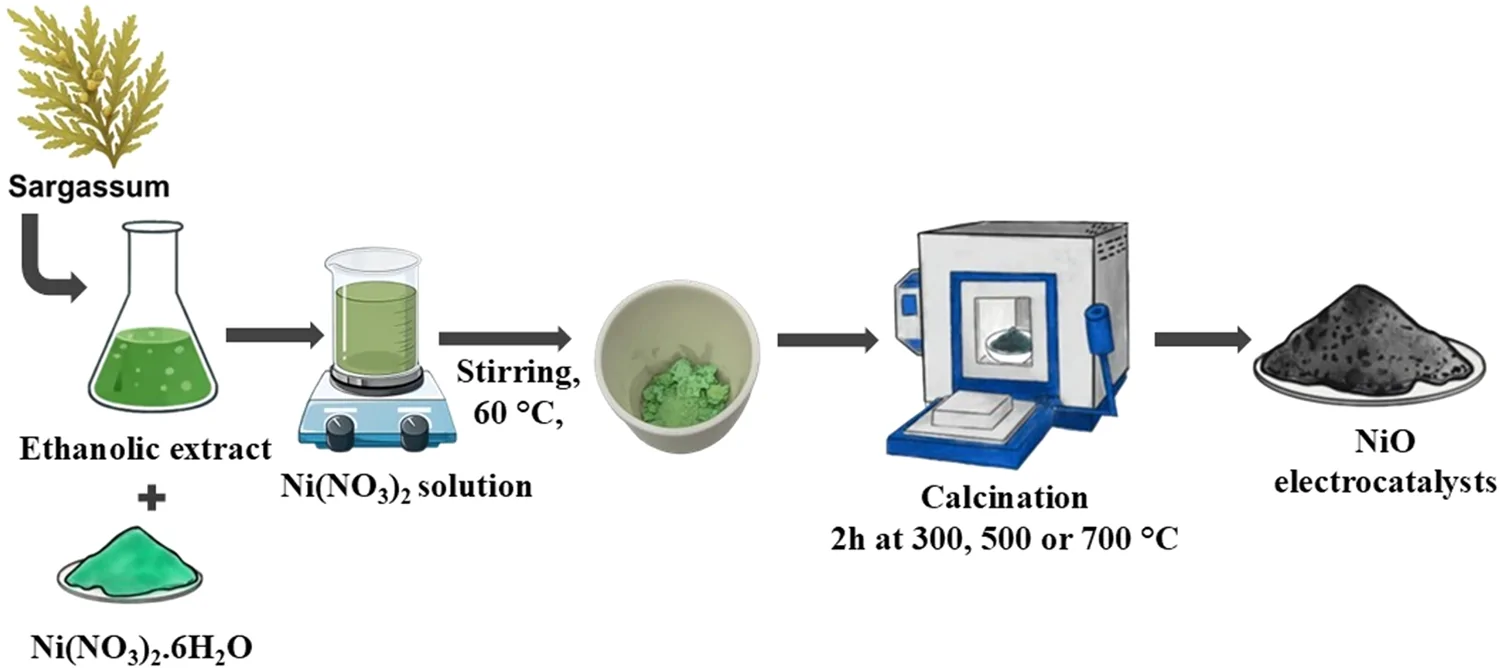
Illustration of the experimental procedure. The graphical illustration was prepared with the assistance of ChatGPT (OpenAI) and subsequently reviewed and edited by the authors.

### 2.5 Samples Characterization

The sample phase was obtained through X-ray powder diffraction using Cu Kα radiation (λ = 0.154 nm) on a SmartLab SE diffractometer (Rigaku, Japan). The data were recorded with a step size of 0.02° and a scan rate of 2° min^–1^. The program Fullprof Suite was employed for refining the XRD data using the Rietveld method, using the CIF file 9866-ICSD as reference. The bottom fitting and peak profiling were performed using a 6-coefficient polynomial function and the Thompson-Cox-Hastings pseudo-Voigt function with axial divergence asymmetry, respectively. In the refinement analysis, the refined parameters were Scale factor, background, lattice parameters, profile half-width parameters (u,v,w), atomic positions, isotropic thermal parameters, and occupancy. Infrared (FTIR) spectra (4000–400 cm^–1^) in KBr pellets were recorded using a Shimadzu IRSpirit spectrophotometer. Raman spectroscopy measurements were carried out at room temperature using an XploRA PLUS spectrometer (HORIBA) equipped with a 532 nm laser (1.75 mW) as the excitation source. TGA data were acquired in a Shimadzu TGA-60/60H thermogravimetric analyzer with alumina sample holders. Experiments were performed under a synthetic air flow (50 mL min^–1^) with a heating rate of 10 °C min^–1^ up to 800 °C. UV–Vis absorption spectra were measured using a Shimadzu UV-2600i spectrophotometer. For morphological characterization, the samples were coated with a thin layer of gold using a Quick Coater SC-701 metallizer (Sanyu Electron). SEM images were acquired using a TESCAN MIRA3 microscope equipped with a tungsten filament and an energy-dispersive X-ray spectroscopy (EDS) detector, operating at an accelerating voltage of 15 to 25 keV. X-ray photoelectron spectroscopy was acquired using an XPS spectrometer (ScientaOmicron ESCA+) with monochromatic Al K_α_ radiation (*h*ν = 1486.6 eV). High-resolution XPS spectra were obtained at a constant energy pass of 20 eV and energy step of 0.05 eV, and the data processing was performed using the CasaXPS software. Room-temperature continuous-wave electron paramagnetic resonance (EPR) measurements were conducted at an X-band (9.53 GHz), with a modulation frequency of 100 kHz using a Bruker Magnettech ESR500 spectrometer. For the measurements, 5 mg of sample was placed into a 4 mm-diameter quartz tube and transferred to the EPR cavity. The spectra were acquired under 0.5 mT.

### 2.6 OER Measurements

Working electrodes were prepared by depositing the electrocatalyst onto precleaned nickel foam substrates (1 cm^2^) using hydrochloric acid. Then, 5 mg of electrocatalyst was dispersed in a solution of 50 μL Nafion and 500 μL isopropyl alcohol, followed by ultrasonication to ensure homogeneity. The suspensions were dropped onto the nickel foam and dried at room temperature under ambient conditions. The active mass of the electrodes averaged 4.7 mg. OER electrocatalytic performance was investigated using a Metrohm Autolab PGSTAT204 potentiostat/galvanostat equipped with a FRA32 M impedance module. Electrochemical measurements were performed at room temperature in alkaline (1.0 mol L^–1^ KOH, pH 13.6). The electrochemical cell consisted of a Pt plate (1 cm^2^) counter electrode, an Ag/AgCl (saturated) reference electrode, and NiO-based working electrodes. Initial OER activity was evaluated via linear sweep voltammetry (LSV, 5 mV·s^–1^) and cyclic voltammetry (CV, 10–100 mV·s^–1^). Electrochemical impedance spectroscopy (EIS) was conducted in the frequency range of 0.1 Hz to 10 kHz, amplitude of 10 mV, at three applied potentials (1.50, 1.55, and 1.60 V vs RHE). All data were corrected for ohmic resistance (*iR* drop) and converted to the reversible hydrogen electrode (RHE) scale using the Nernst equation: *E*_RHE_ = *E*_measured_ + (0.059·pH) + 0.1976. The overpotential (η) was calculated as η = *E*_RHE_ – 1.23 V.

## 3 Results and Discussion

### 3.1 Green Synthesis of NiO

To facilitate the discussion of the structural evolution of synthesized materials, Figure 2 illustrates the proposed mechanism for the formation of NiO nanoparticles mediated by the *Sargassum* sp. extract. Although the individual molecular events cannot be directly verified under the present experimental conditions, the proposed pathway is consistent with previous reports on phytochemical-mediated synthesis of metal oxide nanoparticles.^25,26^ *Sargassum* species are naturally rich in alginates, fucoidans, phlorotannins, proteins and other oxygenated metabolites containing hydroxyl, carboxyl, carbonyl, ether and sulfate groups, which provide multiple coordination sites for Ni^2+^ ions.^29^ These phytochemicals are generally recognized as coordinating, stabilizing and capping agents that promote the formation of Ni-organic complexes while regulating nanoparticle nucleation and growth. However, because the composition of biological extracts varies considerably, the exact molecular pathway remains system-dependent and has not yet been fully elucidated.^41,42^

**Figure 2:**
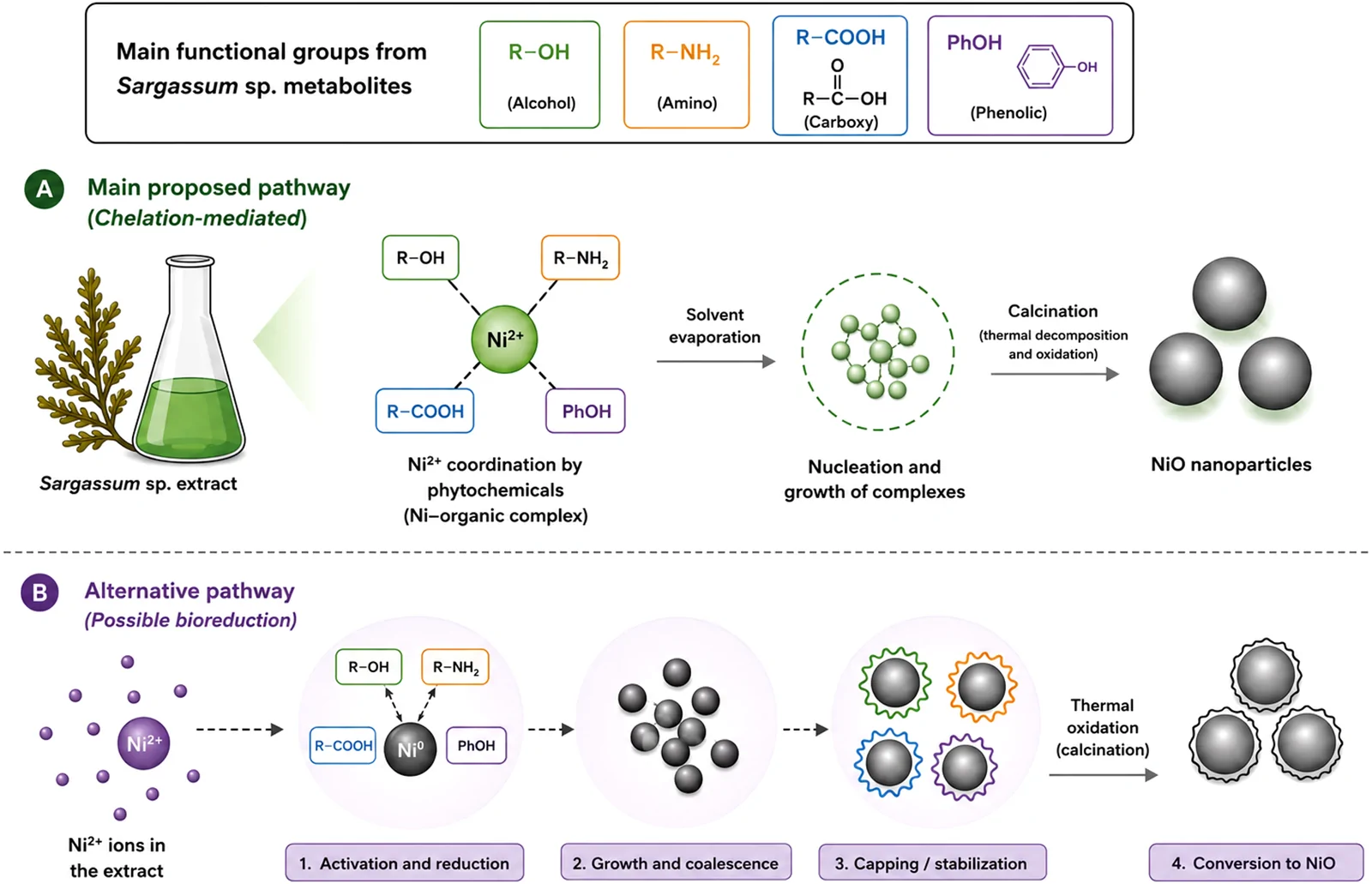
Proposed mechanism for the formation of NiO nanoparticles mediated by the ethanolic extract of *Sargassum* sp. The graphical illustration was prepared with the assistance of ChatGPT (OpenAI) and subsequently reviewed and edited by the authors.

After the addition of nickel nitrate to the ethanolic *Sargassum* extract, Ni^2+^ ions are likely coordinated by oxygen-rich phytochemicals, giving rise to dispersed Ni-organic complexes that act as molecular precursors for nanoparticle formation. Similar coordination-mediated pathways have been proposed for the green synthesis of NiO, where phenolic compounds chelate Ni^2+^ ions before undergoing thermal conversion into crystalline oxide.^43^ As solvent evaporation proceeds, the concentration of these coordinated species increases, may promoting homogeneous nucleation, while the surrounding organic matrix limits excessive particle growth and agglomeration through steric stabilization. During calcination, the organic components are progressively decomposed, whereas the nickel-containing intermediates undergo dehydration and oxidation, resulting in the crystallization of the NiO phase. Therefore, in the present synthesis, the *Sargassum* extract acts primarily as a multifunctional reaction medium that coordinates Ni^2+^ ions, stabilizes intermediate species and directs nanoparticle formation prior to thermal conversion. It should be noted that an alternative mechanism involving the phytochemical-mediated reduction of metal ions has been widely proposed for the biosynthesis of noble metal nanoparticles, particularly Ag and Au.^33,44^ Whether a similar reduction pathway occurs during the green synthesis of transition metal oxides remains controversial. Therefore, although a possible bioreduction route is illustrated in Figure 2 for completeness, the coordination-mediated pathway is considered the predominant mechanism for NiO formation in the present system, in agreement with previous reports on phytochemical-assisted synthesis of nickel oxide nanoparticles.^25^

### 3.2 Characterization of the Electrocatalysts (NiO-300, NiO-500, and NiO-700)

Figure S1 shows the X-ray diffractogram patterns of NiO samples. As expected, the NiO-300, NiO-500, and NiO-700 samples exhibited a cubic symmetry and *Fm*3̅*m* space group (ICSD-9866), agreeing with other reports in the literature.^45^ Figure 3 shows the Rietveld refinement, confirming the cubic structure of the NiO-300 sample. From this diffraction data, both the size and strain have been calculated. Initially, the size from Scherrer’s method was calculated, where the XRD peak has broadened in the nanocrystals due to the crystalline size effect and intrinsic strain effect. So, the peak broadening normally consists of two parts: physical broadening and instrumental broadening.^46^ The average crystallite size calculated was 16.22, 23.22, and 34.02 nm for the NiO-300, NiO-500, and NiO-700 samples, respectively. For the NiO­(w) sample obtained in the absence of seaweed extract, XRD data (Figure S2) confirms the formation of phase-pure NiO (crystallite size 19.72 nm, *a* = 4.17967 Å).

**Figure 3:**
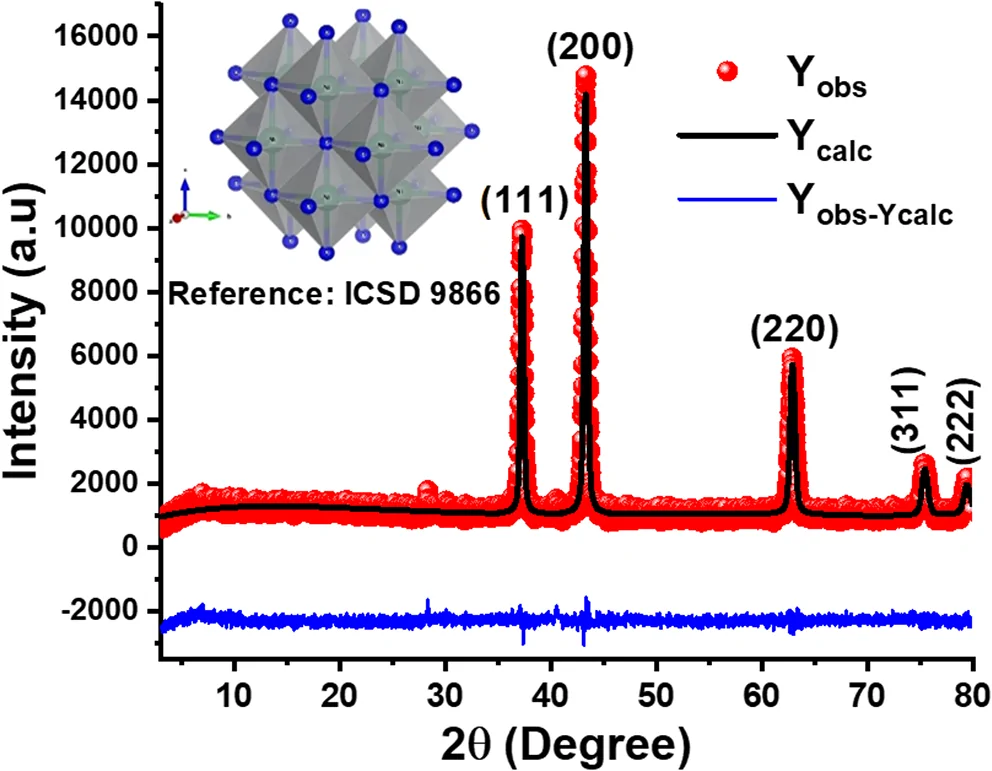
XRD pattern (red ball), Rietveld refinement (black line), and *Y*_obs_– *Y*_calc_ (blue line) for NiO-300 sample. The inset shows the cubic structure of NiO.

It is known that Scherrer’s method considers only one effect of crystallite size on the peak broadening and does not say anything about the intrinsic strain due to the grain boundary and defects. To resolve this question, it was applied for the *Uniform Deformation Model* (*UDM*) was applied to estimate the microstrain (ε). For this work, we have used the Williamson-Hall approach given by

$$\beta_{\mathrm{hkl}}\cos\theta =\frac{K\lambda }{D}+4\epsilon \sin\theta$$

where: β_hkl_ is full width at half of the maximum intensity, θ is the Bragg angle, *K* is a constant, λ is the wavelength of the incident X-ray (λ = 0.154 nm), and *D* is the crystallite size.^47^ The β_hkl_ cos θ × 4 sin θ were plotted for the NiO-300, NiO-500, and NiO-700 samples. From the plot, the crystallite sizes obtained were 92.20, 35.83, and 77.00 nm, respectively. The result obtained shows a big difference between the values obtained from the Scherrer method and an inconsistency; the sample NiO-500 shows a smaller crystallite size. This result cannot occur due to the NiO-500 being calcinated at a temperature higher than NiO-300, which indicates the Williamson-Hall approach is not a better method for them to calculate the crystallite size and microstrain. In this sense, the crystallite size and microstrain have now been evaluated using the Size-Strain plot (SSP). In this method, it is assumed that the “strain profile” is explained by a Gaussian function and the “crystallite size” profile by a Lorentzian function given by the equation

$${\left(d_{\mathrm{hkl}}\beta_{\mathrm{hkl}}\cos\theta \right)}^{2}=\frac{K\lambda }{D}\left(d_{\mathrm{hkl}}^{2}\beta_{\mathrm{hkl}}\cos\theta \right)+\frac{\epsilon^{2}}{4}$$

where the *d*_hkl_ is the lattice distance between the (hkl) planes.^48^ The plot of (*d*_hkl_^2^β_hkl_ cos θ) × (*d*_hkl_β_hkl_ cos θ)^2^ for the NiO-300, NiO-500 and NiO-700 samples reveal that crystallite size was approximately 48.42, 33.63 and 50.56 nm, respectively. Another possible way to measure the crystallite size and microstrain is by using the Halder-Wagner method. For this method the peak broadening is well adjusted with the Voigt function.^49^ Now the relation between the crystallite size and microstrain is given by

$${\left(\frac{\beta_{\mathrm{hkl}}^{*}}{d_{\mathrm{hkl}}^{*}}\right)}^{2}=\frac{1}{D}\cdot \frac{\beta_{\mathrm{hkl}}^{*}}{{d_{\mathrm{hkl}}^{*}}^{2}}+{\left(\frac{\epsilon }{2}\right)}^{2}$$

where: β_hkl_^*^ = β_hkl_ cos θ/λ and *d*_hkl_^*^ = 2*d*_hkl_ sin θ/λ. The plot of $\left(\frac{\beta_{\mathrm{hkl}}^{*}}{{d_{\mathrm{hkl}}^{*}}^{2}}\right)\times {\left(\frac{\beta_{\mathrm{hkl}}^{*}}{d_{\mathrm{hkl}}^{*}}\right)}^{2}$ for the NiO-300, NiO-500 and NiO-700 samples reveal that the crystallite size was approximately 100.07, 155.62 and 222.27 nm. So, the Halder-Wagner method has shown the ideal model for the crystallite size for the samples worked here, which indicates an increase in the crystallite size with the increase of calcination temperature. Table S1 shows the crystallite size (*D*) in nm and microstrain (ϵ) calculated by Scherrer, Williamson-Hall, Size-Strain plot (SSP), and Halder-Wagner (H-M) plot. Table S2 shows the lattice parameter in (Å) and χ^2^.

The FTIR spectrum of the NiO samples is shown in Figure 4(a). The NiO-300 sample calcined at 300 °C exhibited an absorption band near 3577 cm^–1^, attributed to the O–H stretching vibration of hydroxyl groups on the NiO surface.^50^ Bands at 1628, 1529, and 1384 cm^–1^ correspond to the stretching vibrations of C=O, C=C, and C–O bonds, while bands at 1308 and 720 cm^–1^ are associated with C–H bending vibrations, and the band at 972 cm^–1^ with C=C deformation. These functional groups are assigned to residual organic groups. Additionally, an absorption band related to the stretching vibration of the metal–oxygen (Ni–O) bond was also observed at 441 cm^–1^.^45^ The FTIR spectra of NiO-500 and NiO-700 displayed bands related to Ni–O stretching. However, their FTIR spectra showed a notable decrease in the intensity of peaks associated with organic functional groups, which is due to calcination at higher temperatures.^48^

**Figure 4:**
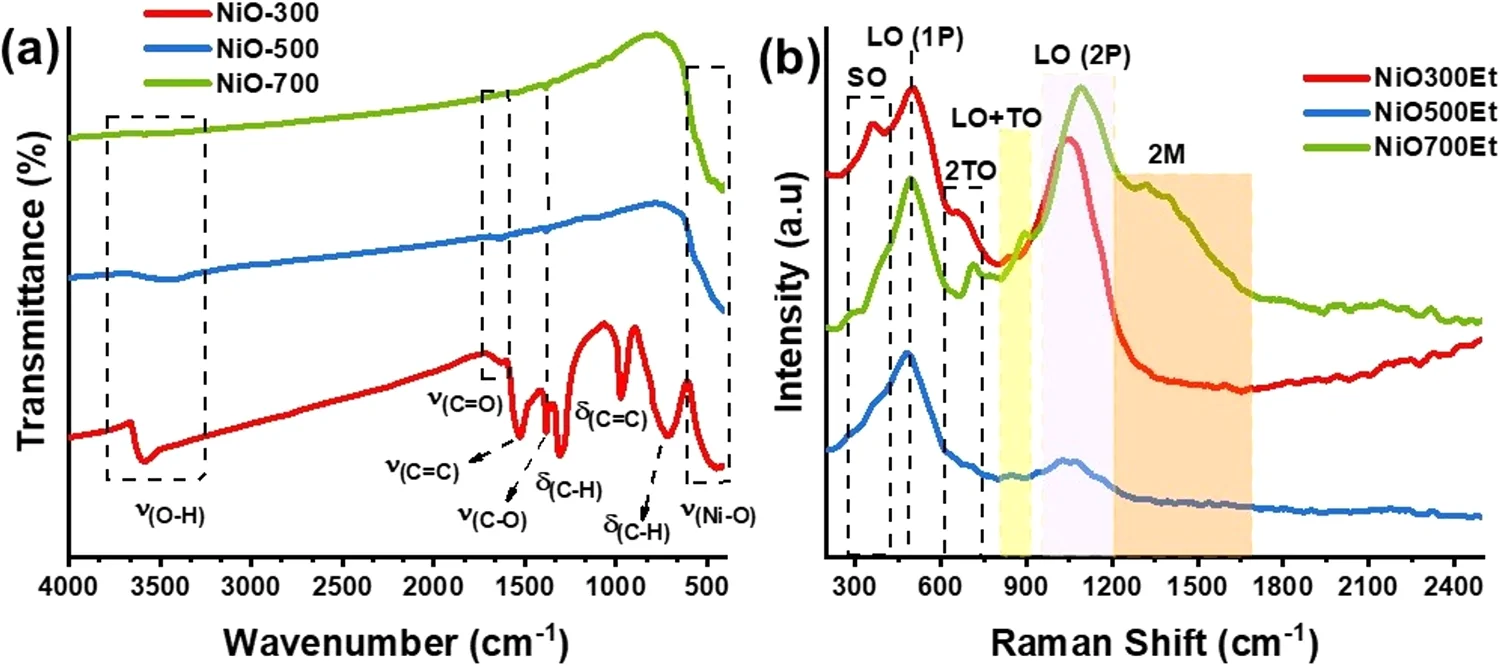
(a) Infrared and (b) Raman vibrational spectra of the NiO samples.

The vibrational spectra obtained by Raman scattering (Figure 4(b)) revealed characteristic bands linked to one-phonon optical modes (SO, TO, LO), two-phonon modes (2TO, TO + LO, LO­(2P)), and two-magnon (2M), consistent with previously reported literature data.^45,49–52^ The surface optical phonon mode (SO) can be overlapped by the longitudinal optical modes (LO) in the low wave vector region (*q* ≈ 0).^53,54^ However, activating SO modes indicate a break in surface symmetry, often linked to the roughness or shape of the nanostructure.^55^ Nanoparticles with a rectangular shape tend to be more sensitive to surface symmetry, which enhances the observation and strength of these modes.^55,56^ The Raman spectrum of NiO calcined at 300 °C (Figure 4(b)) shows a stronger signal related to the SO mode compared to samples calcined at higher temperatures. This behavior is likely due to its mainly rectangular shape, whereas the samples heat-treated at higher temperatures have a spheroidal form, as observed in a scanning electron microscope (SEM). The first-order longitudinal optical phonon (LO(1)) shows an intense signal in the samples. However, this mode only becomes Raman-active due to the presence of structural defects or distortions in the rhombohedral or cubic NiO lattice.^57^ The second-order longitudinal optical phonon (LO(2)) mode is associated with Ni^2+^ vacancies.^58,59^ These bands showed greater intensity in the NiO-700 sample, with a shift to the higher energy region (blue shift). In contrast, the LO(1) intensity decreases, which is qualitatively consistent with changes in surface defect-related species in XPS analyses. The Raman signals corresponding to the two-phonon modes, such as the optical transverse (2TO) and the optical longitudinal-transverse combination (LO + TO), are associated with the paramagnetic phase of NiO.^56^ The presence of two magnon (2M) bands is related to the Ni^2+^–O^2^–Ni^2+^ superexchange interaction.^56,60^ The higher intensity observed in the NiO-700 sample may be attributed to the increase in calcination temperature, which leads to the formation of larger particles.

Figure S3 presents the optical properties of NiO obtained by solid-state UV–Vis spectroscopy. In Figure S3­(a), characteristic absorption bands associated with Ni^2+^ electronic transitions in an octahedral symmetry (*O_h_*) environment are observed. In the higher-energy UV region, an intense absorption band is detected and attributed to ligand-to-metal charge transfer (LMCT), specifically O^2–^ → Ni^2+^, followed by less intense bands in the visible region corresponding to electronic transitions from the ground state ^3^A_2g_ to the excited states ^3^T_2g_ (F), ^3^T _1g_ (F), and ^3^T _1g_ (P).^60,61^ Figures S3­(b–d) show the band gap values obtained using the Tauc method. The NiO-300, NiO-500, and NiO-700 samples yielded values of 3.23, 3.41, and 3.28 eV, respectively. These values agree with the energy range reported in the literature.^39,62,63^ Thermogravimetric analysis (TGA) was performed to detect organic residues in the samples, and the results are shown in Figure S4. The NiO-300 sample showed the highest mass loss, approximately 15.1%, which is attributed to organic residues and water absorbed on its surface, as also observed in FTIR data (Figure 4(a)). This behavior results from the low temperature and short calcination time, which were inadequate to remove the organic compounds. The NiO-500 and NiO-700 samples showed lower mass losses of 2.6 and 2.1%, respectively, which indicates a lower presence of adsorbed water on the surface.

The scanning electron microscope (SEM) (Figures 5 and S5) shows the surface morphology of NiO samples calcined at various temperatures. In Figure S5­(a), which corresponds to NiO calcined at 300 °C, an agglomerate of particles with a cubic shape and relatively regular surface can be seen, indicating controlled growth. Figure S5­(b), related to NiO calcined at 500 °C, shows agglomerates made up of smaller particles with a spheroidal shape. The temperature increase promotes a particle coarsening and morphological reorganization of the material, leading to the observed changes in morphology. In Figure S5­(c), corresponding to NiO calcined at 700 °C, there is a notable increase in particle size, likely due to coalescence caused by the high calcination temperature, which encourages particle growth and fusion.

**Figure 5:**
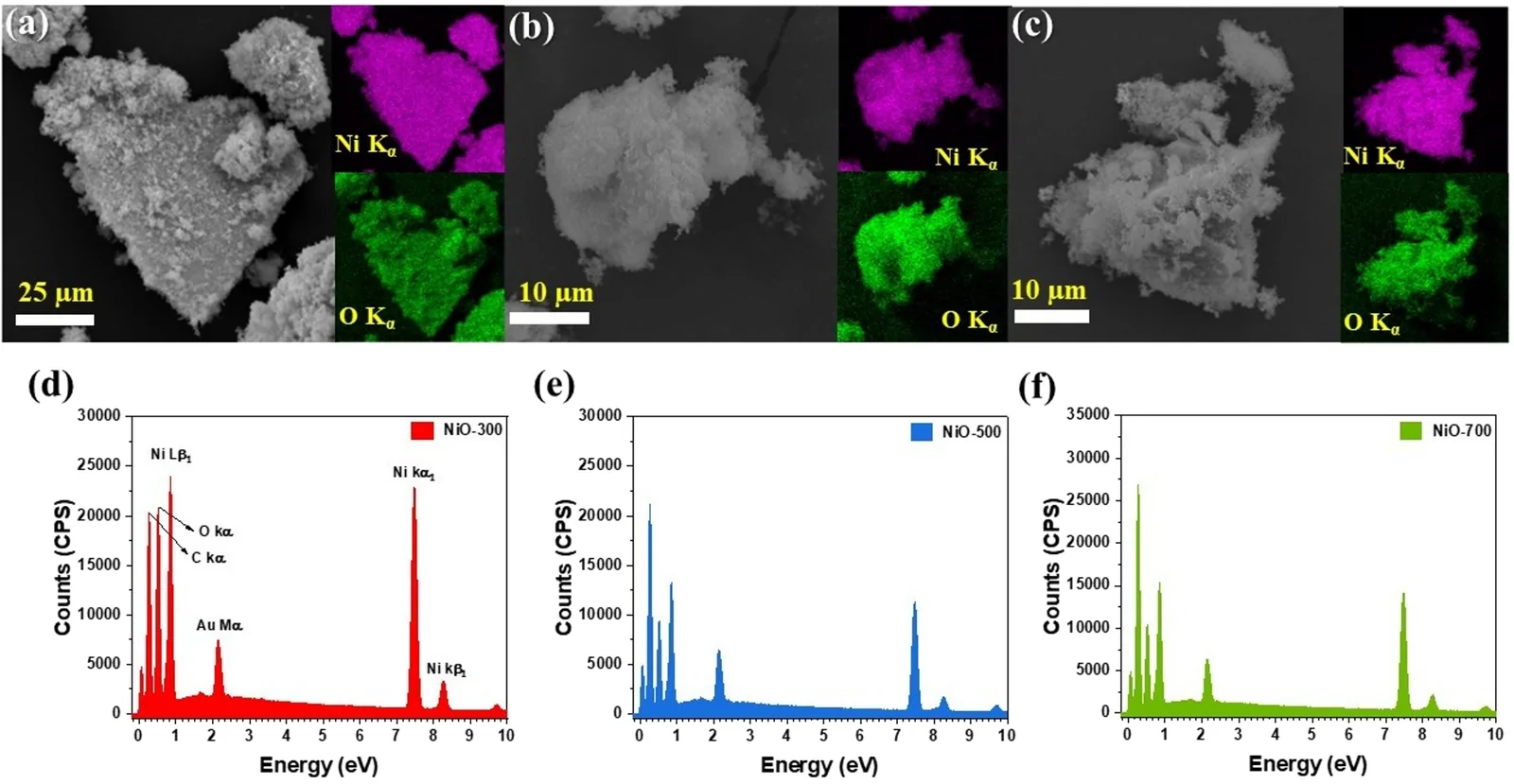
SEM images with EDX elemental mapping of NiO-300, NiO-500, and NiO-700: (a–c) distribution of Ni (Ni Kα) and O (O Kα); (d–f) corresponding EDX spectra.

Additionally, the average particle size distribution was analyzed for the NiO-500 and NiO-700 samples in Figure S5­(d,e), which had average particle sizes of 74.75 and 237.06 nm, respectively. For the NiO-300 sample, it was not possible to perform size distribution analysis due to the cubic shape of the particles, which hinders the application of the conventional statistical method. EDS analysis, also presented in Figure 5, shows the spectra obtained for the NiO-300, NiO-500, and NiO-700 samples. The elemental mapping images (Figure 5(a–c)) reveal a homogeneous distribution of Ni (Ni Kα) and O (O Kα) across the analyzed regions, indicating that both elements are uniformly dispersed throughout the samples without noticeable segregation. The EDX spectra shown in Figure 5(d–f) were used to analyze the elemental composition of the NiO-300, NiO-500, and NiO-700 samples. In all cases, only peaks corresponding to nickel and oxygen were observed, confirming the expected chemical composition of the materials, with no evidence of detectable impurities.

X-ray Photoelectron Spectroscopy (XPS) measurements were performed for the NiO samples obtained after calcination at different temperatures. The survey XPS spectra (Figure 6) revealed the presence of Ni, O, and C elements on the surface of the samples with slightly different elemental concentrations as indicated in Table S3. Deconvolution of the high-resolution (HR) XPS spectra was conducted using CasaXPS software. All the spectra were fitted using a Shirley background, and Lorentzian–Gaussian (GL) line shape, and the spectra are shown in Figure 6(b–d). The HR Ni 2p_3/2_ XPS spectra of various samples are shown in Figure 6(b), which consists of multiplet-split components. It is worth noting that no significant changes in the spectral features or peak positions were observed, indicating that the target composition was successfully achieved in the samples. The spectral deconvolution of the Ni 2p_3/2_ spectra originates from the doublet peaks and overlapping satellite peaks of Ni species, characteristic of NiO.^64–69^ Mixed Ni­(II) and Ni­(III) species were identified in the samples, which agrees with the work reported by Sagui et al.^70^ Higher Ni­(III) content was observed when compared to Ni­(II) species, possibly due to the nonstoichiometry of NiO samples.^71^ It is important to note that Ni^3+^ ions are the common intrinsic point defects formed via the charge-compensation mechanism in nonstoichiometric NiO samples,^72^ which play an essential role in the OER performance.

**Figure 6:**
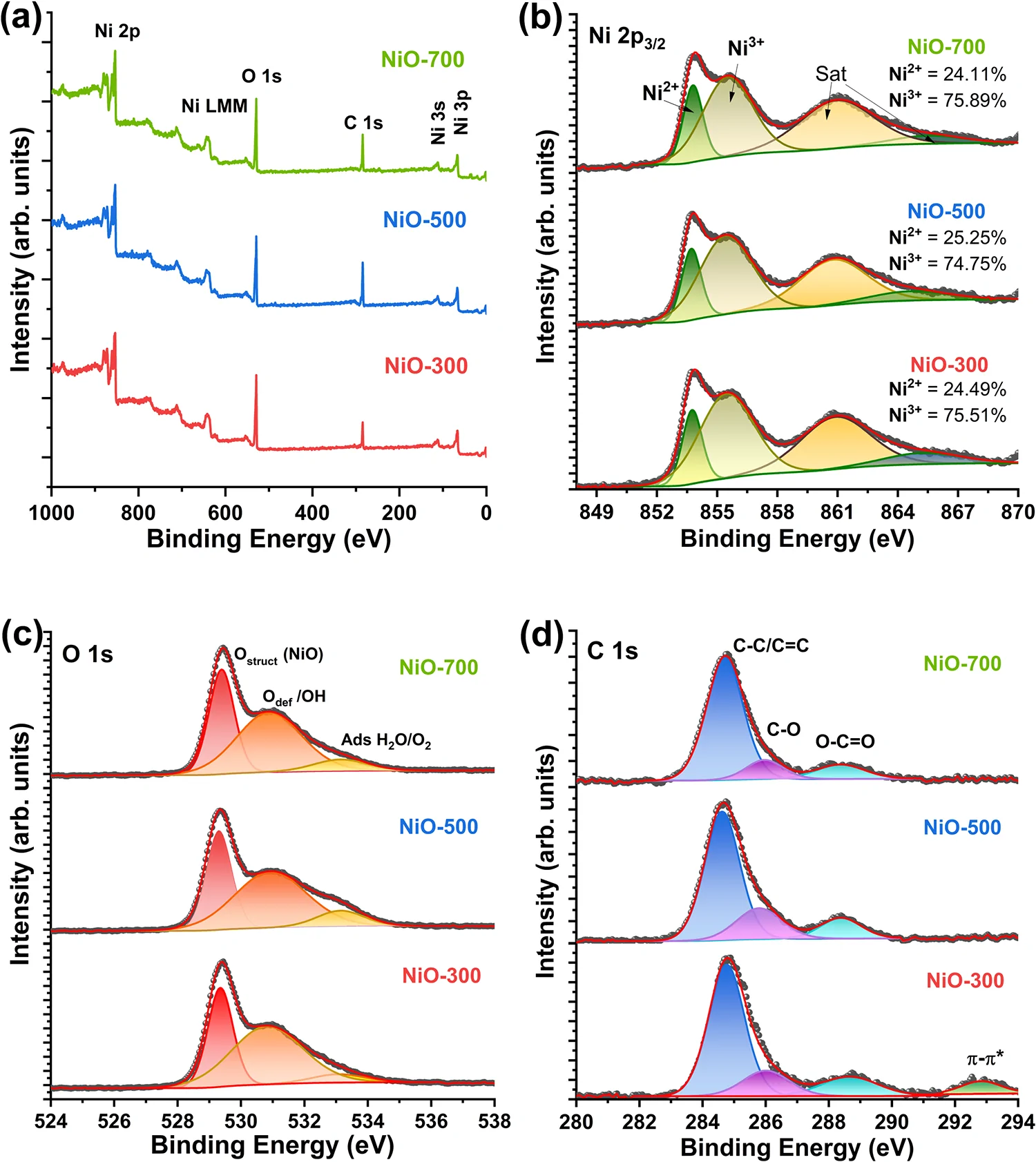
Survey spectra (a), High-resolution of Ni 2p (b), O 1s (c), and C 1s (d) of the NiO samples.

The Ni­(II) and Ni­(III) peaks appear at the binding energies of approximately 853.71–853.78 and 855.42–855.47 eV, respectively. The binding energy separation (Δ*E*) was in the range of 1.68–1.72 eV in the samples (Table S4), consistent with prior works on NiO.^67^ Despite this, several authors have not differentiated Ni­(II) and Ni­(III) species in NiO samples. The BE values estimated by the deconvolution analysis of Ni 2p_3/2_ spectra are close to those reported for NiO and Ni_2_O_3_, considering the error of the values in chemical states of about 0.5 eV.^73^ Confirming the presence of Ni­(II) and Ni­(III) in the samples synthesized in the present work. According to the literature, the presence of Ni­(III) results from the nickel deficiency in the samples. This may also lead to the formation of NiOOH rather than the Ni_2_O_3_ phase,^74^ which is consistent with current XRD data. The surface chemical concentrations of Ni­(II) and Ni­(III) were then determined from the XPS deconvolution analysis, as listed in Table S4. According to the XPS fitting (Figure 6(b) and Table S4), a higher amount of Ni­(III) was present on the surface, reaching 75–76% in the samples. The Ni­(II) and Ni­(III) concentrations in the samples are indicated in Figure 6(b).

Deconvolution of the HR O 1s XPS spectra reveals three peaks at binding energies between 529.30 and 533.13 eV (Figure 6(c), Table S5), which originate from NiO, hydroxyl groups in NiOOH or O-related defects, and adsorbed species. The first peak, at 529.30–529.39 eV, corresponds to bound structural oxygen in the NiO lattice. The second peak, at 530.82–530.93 eV, might be attributed to Ni­(III)-O bonds or excess oxygen from atoms in the NiO lattice (NiOOH). Particularly, resulted from the hydroxylated species that form a NiOOH surface containing Ni­(III), as indicated by Ni 2p XPS (Figure 6(b)), consistent with data reported by Sabat et al.^71^ and Barreca et al.^74^ Some researchers have assigned this to OH groups on oxygen-deficient sites.^72,75^ The final O 1s peak, at 533.06–533.13 eV, may result from adsorbed H_2_O or O_2_ molecules. Apart from the Ni 2p and O 1s signals, the presence of C 1s represents the residual carbon remaining after calcination. The C 1s spectrum is shown in Figure 5(d), and the BE positions are listed in Table S6. The deconvoluted C 1s spectra were fitted assuming contributions from allylic and aliphatic carbon appearing at BE between 284.62 and 284.76 eV, the other peaks at approximately 285.78–286.01 eV, 288.37–288.67, and 292.82 are assigned to C–O/C=O, COOH/COOR, and π–π*, respectively.^76^

To provide further information on the defect formation in NiO samples, EPR spectroscopy was performed as shown in Figure 7. The EPR revealed a broad resonance associated with Ni^2+^ ([Xe] 3d^8^, *S* = 1) species due to strong spin–spin interactions, characteristic of antiferromagnetic NiO.^72,75^ This broadband is larger for the NiO-700, calcined at 700 °C, indicating a greater contribution from Ni^2+^ and stronger spin–spin interactions. Apart from this, a weak and narrow EPR resonant signal at approximately *g* = 2.0005–2.0006 is observed, which is related to the oxygen vacancy (V_O_^•^/ F^+^ centers),^72^ which agrees with XPS data. This signal intensity is higher in the NiO-300 and NiO-500 samples, indicating a higher Vo concentration than in NiO-700. Additionally, despite the increased concentration of Ni^3+^ species observed in XPS at the surface, no evident signal corresponding to these species has been observed in the current EPR spectra. The absence of a well-resolved signal of Ni^3+^ species may indicate that these species are essentially on the surface, with lower concentrations in the bulk, whose signals may be superimposed on the broad resonance in the EPR spectra.

**Figure 7:**
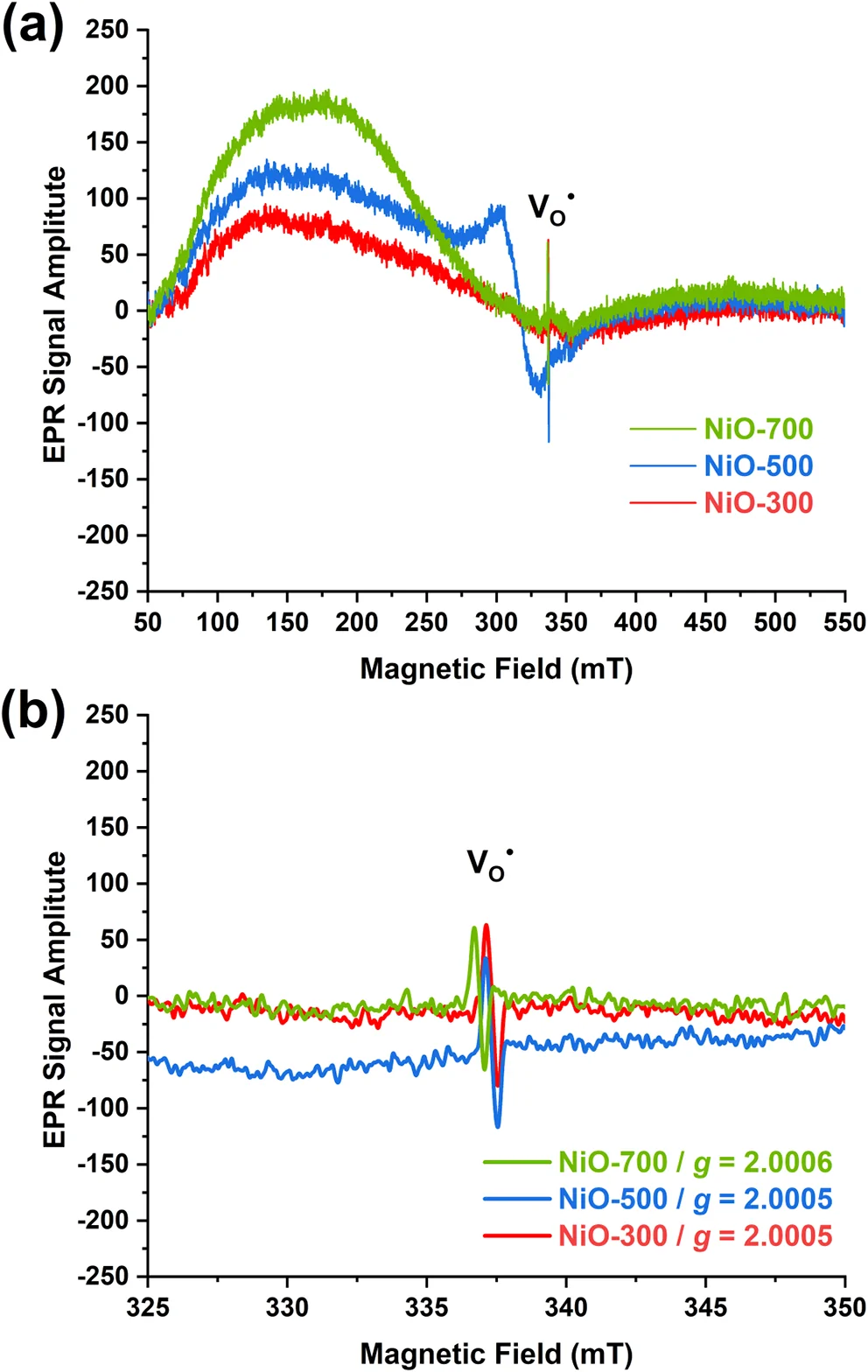
Electron paramagnetic resonance (EPR) spectra of NiO-300, NiO-500, and NiO-700. (a) Full EPR spectra. (b) Expanded view of the signal attributed to oxygen-vacancy-related *F* ^+^centers (*g* ≈ 2.0005 – 2.0006).

### 3.3 Electrochemical Performance

The electrocatalytic performance of the NiO samples toward the OER was investigated by linear sweep voltammetry (LSV) in 1.0 mol/L KOH aqueous solution, as presented in Figure 8(a). A distinct anodic feature observed at approximately 1.34 V is attributed to the oxidation of surface Ni^2+^ species and the formation of NiOOH-like Ni^3+^ species, which are commonly associated with the catalytically active phase of Ni-based oxides under alkaline OER conditions. The η values required to achieve a current density of 10 mA cm^–2^ were 352 mV for NiO-300, 368 mV for NiO-500, and 356 mV for NiO-700. By comparison, bare Ni foam and the control sample synthesized without the algae extract (NiO­(w), Figure S6) required considerably higher overpotentials of 514 and 392 mV, respectively. The lower η values observed for the algae-mediated NiO samples indicate that the green synthesis strategy effectively enhances the electrocatalytic performance toward the OER, with NiO-300 exhibiting slightly higher activity among the investigated catalysts. According to the classification proposed by Tahir et al., these values can be considered indicative of excellent electrocatalytic performance.^77^

**Figure 8:**
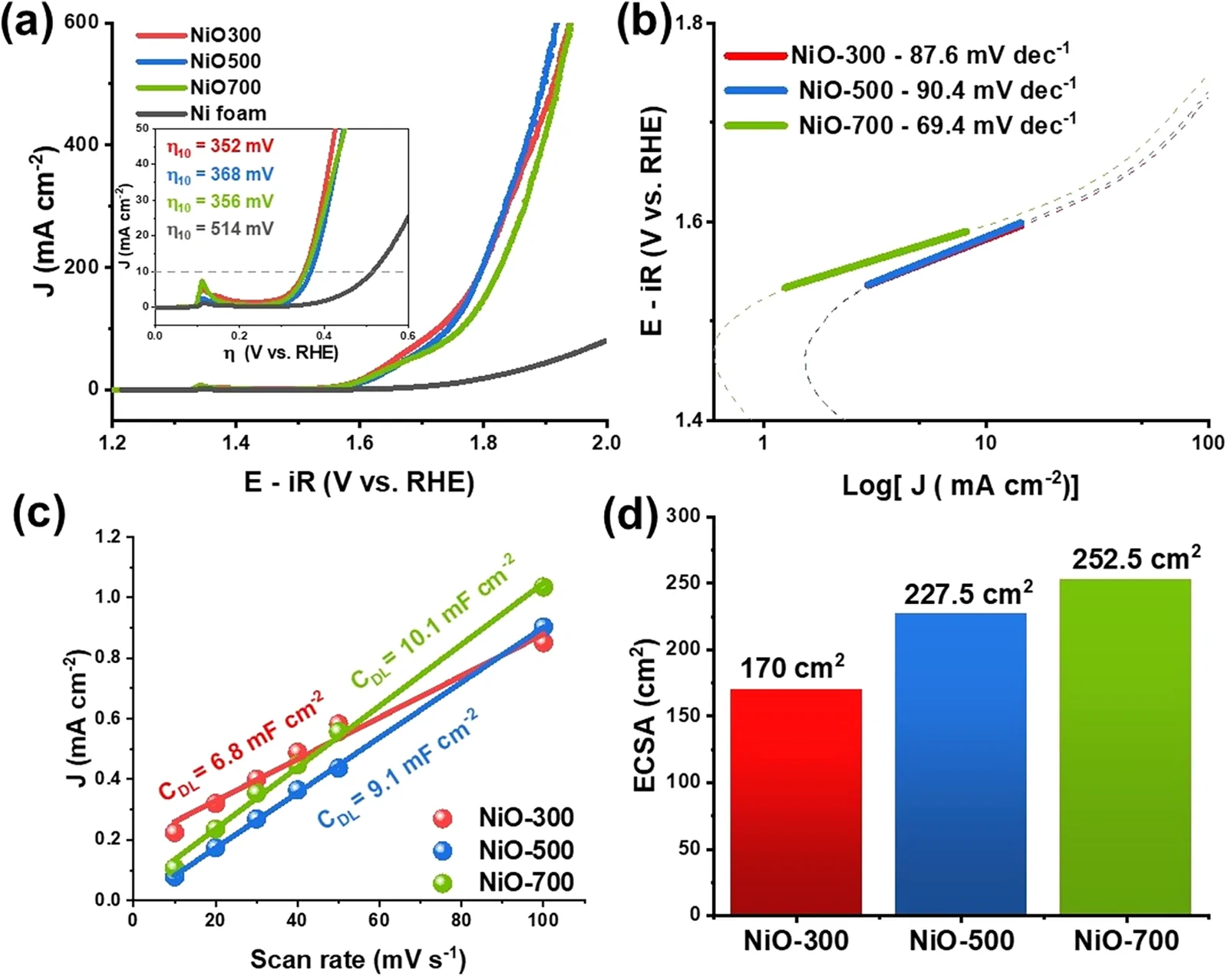
(a) Anodic polarization curves obtained from LSV showing the overpotential for each prepared electrode; (b) corresponding Tafel plots; (c) double-layer capacitance (*C*_DL_) determined from CV measurements; and (d) electrochemically active surface area (ECSA).

The OER kinetics were evaluated using Tafel plots (Figure 8(b)), derived from the relationship: *η = a + b *log*(J*_0_ *)*, where η represents the overpotential, *a* is the intercept associated with the exchange current density (*J*_0_), and *b* corresponds to the Tafel slope. The obtained Tafel slope values were 87.6 mV dec^–1^, 90.4, and 69.4 mV dec^–1^ for NiO-300, NiO-500, and NiO-700 samples, respectively. According to the conventional adsorbate evolution mechanism (AEM), originally described by the classical Krasil’shchikov model for the four-step OER in alkaline media (Reactions 1–4), each elementary step is associated with a characteristic Tafel slope.^78^ The Tafel slopes obtained for all samples are closer to the value expected for the second elementary step (M*OH + OH^–^ → M*O^–^ + H_2_O), suggesting that the second elementary step may play an important role in the reaction kinetics if the OER proceeds predominantly through the conventional adsorbate evolution mechanism. Notably, the NiO-700 sample exhibited the lowest Tafel slope (69.4 mV dec^–1^), indicating more favorable reaction kinetics than the other samples.

1 $$M^{*}+OH^{-}\to M^{*}\mathrm{OH}+e^{-},,b=120\;\mathrm{mVde}c^{-1}$$

2 $$M^{*}\mathrm{OH}+OH^{-}\to M^{*}O^{-}+H_{2}O,,b=60\;\mathrm{mVde}c^{-1}$$

3 $$M^{*}O^{-}\to M^{*}O+e^{-},,b=45\;\mathrm{mVde}c^{-1}$$

4 $$2M^{*}O\to 2M^{*}+O_{2},,b=19\;\mathrm{mVde}c^{-1}$$

The anodic feature observed before the onset of OER is attributed to the oxidation of surface Ni^2+^ species into NiOOH-like Ni^3+^ species, which are widely recognized as the catalytically active phase of Ni-based electrocatalysts in alkaline media. Under anodic polarization, NiO surfaces may undergo hydroxylation and partial surface reconstruction, resulting in an active surface that differs from the as-prepared material.^79^ Within the conventional adsorbate evolution mechanism (AEM), the OER proceeds through the sequential adsorption and transformation of *OH, *O, and *OOH intermediates before O_2_ evolution. The Tafel slopes obtained in this work are broadly consistent with a significant contribution from the second elementary step of the AEM, although Tafel analysis alone cannot unambiguously identify the reaction pathway.

Raman and XPS analyses revealed defect-related structural and surface species, while EPR spectroscopy provided additional evidence of oxygen-vacancy-related *F* ^+^centers, with a more intense signal for NiO-300 and NiO-500 than for NiO-700. These oxygen vacancies may modify the local Ni–O electronic structure, facilitate surface hydroxylation and the formation of highly oxidized Ni species, and influence the adsorption energetics of OER intermediates. Such effects, together with the lower charge-transfer resistance of NiO-300, may contribute to its enhanced electrocatalytic activity. However, direct confirmation of lattice-oxygen participation, potential-dependent mechanism switching, or other alternative pathways would require operando spectroscopic techniques or isotopic-labeling experiments, which are beyond the scope of the present study.

The double-layer capacitance (*C*_DL_) and the electrochemically active surface area (ECSA) of the NiO samples were estimated from cyclic voltammetry (CV) measurements performed in the nonfaradaic region at scan rates ranging from 10 to 100 mV s^–1^ (Figure S7). The *C*_DL_ values (Figure 8(c)) were determined from the linear dependence of the anodic current (*i*_a_) on the scan rate (*ν*), following the relation *i*_a_ = *C*_DL_ *ν*. Based on this approach, *C*_DL_ values of 6.8, 9.1, and 10.1 mF cm^–2^ were obtained for the NiO-300, NiO-500, and NiO-700 samples, respectively. The higher *C*_DL_ values observed for the NiO-500, and NiO-700 samples indicate an increased electrochemically active surface area (ECSA). The ECSA (Figure 8(d)) was subsequently estimated from the proportional relationship between *C*_DL_ and the specific capacitance (*C*_s_), using the expression ECSA = *C*_DL_/*C*_s_. A value of *C*_s_ = 0.040 mF cm^–2^ was adopted, consistent with typical values reported for metal-based electrodes in aqueous alkaline media.^80^ The calculated ECSA values were 170, 227.5, and 252.5 cm^2^ for the NiO-300, NiO-500, and NiO-700 samples, respectively. Interestingly, although the NiO-300 sample exhibited the lowest overpotential, the NiO-700 sample displayed the highest ECSA. This result suggests that increasing the calcination temperature enhances the exposure of active sites and improves the electrode–electrolyte interaction, while also potentially modifying the intrinsic physicochemical properties of the material.^81^

A comparison with literature (Table S7) reveals that the electrocatalysts synthesized in this work, via a green and cost-effective route, display excellent performance toward the OER. Although some previously reported materials, such as the Ni-NiO/C system described by Zhuo et al., exhibit superior metrics (295 mV at a current and a Tafel slope of 52 mV dec^–1^),^82^ these systems typically rely on more complex synthesis procedures, higher production costs, and the use of less environmentally benign solvents. Table 1 compares the OER performance of the green-synthesized electrocatalysts prepared in this work with representative electrocatalysts obtained using natural extracts reported in the literature. While previous studies have exclusively employed extracts from terrestrial plants, including *Mangifera indica*, *I. asarifolia*, *Aloe vera*, *Olea ferruginea Royle*, *Amaranthus viridis*, and *Pandanus odoratissimus*, the present work is, to the best of our knowledge, the first to report the use of a marine macroalgae (*Sargassum sp.*) extract for the green synthesis of an OER electrocatalyst. The use of Sargassum is particularly attractive because it is an abundant and renewable marine biomass that does not require agricultural land or freshwater resources. In addition, its extract contains polysaccharides, polyphenols, pigments, and other phytochemicals capable of acting as reducing, complexing, and stabilizing agents during nanoparticle formation.

**Table 1:** Comparison of the OER Performance of the Electrocatalysts Prepared in This Work with Green-Synthesized Electrocatalysts Obtained from Plant and Algae Extracts Reported in the Literature

material	η@10 mAcm^–2^ (mV)	Tafel slope (mV dec^–1^)	Plant/algae	solvent	reference
NiO-300	352	87.6	*Sargassum sp.*	Ethanol	this work
NiO-500	368	90.4	*Sargassum sp.*	Ethanol	this work
NiO-700	356	69.4	*Sargassum sp.*	Ethanol	this work
(NiMn_2_O_4_)_et1_	550^a^	180	*M. indica*	Ethanol	^83^
(NiMn_2_O_4_)_et2_	450^a^	131	*M. indica*	Ethanol	^83^
NiO	680^a^	298	*M. indica*	Water	^83^
NiO	620^a^	216	*M. indica*	Ethanol	^83^
Ni300H	307	60.35	*I. asarifolia*	Water	^39^
CuFeO_*x*_	310^b^	46	*A. vera*	Ethanol	^84^
ZrO_2_ nanoparticles	441	71	*Olea ferruginea Royle*	Water	^85^
NiO:ZnO/PdO	410	76	*Olea ferruginea Royle*	Water	^38^
NiO nanoparticles	416	95	*A. vera*	Water	^37^
ZnO/Mn_3_O_4_	427	96	*Olea ferruginea Royle*	Organic solvents	^86^
CuO:La_2_O_3_	351	--	*A. viridis*	Water	^87^
CuO–Bi_2_O_3_	450	141	*A. viridis*	Water	^88^
CuO–ZrO_2_ nanocomposite	430	116	*A. viridis*	Water	^89^
Fe_3_O_4_ nanoparticles	320^c^	295	*P. odoratissimus*	Water	^90^
Mn_3_O_4_ nanoparticles	431	104	*Olea ferruginea Royle*	Water	^91^
Co_3_O_4_	513^b^	259.74	*A. vera*	Water	^92^

a Measured at 100 mA cm^–2^.

b Measured at 50 mA cm^–2^.

c Measured at 1 mA cm^–2^.

From the electrochemical point of view, the NiO catalysts synthesized exhibited competitive OER performance compared with other green-synthesized electrocatalysts reported in the literature. The over potential and Tafel slopes are comparable to, and in several cases lower than, those reported for other green-synthesized materials evaluated under the same current density, including NiO nanoparticles (416 mV), ZrO_2_ nanoparticles (441 mV), NiO:ZnO/PdO (410 mV), ZnO/Mn_3_O_4_ (427 mV), CuO–Bi_2_O_3_ (450 mV), and CuO–ZrO_2_ (430 mV). Although the Ni300H catalyst prepared using *I. asarifolia* showed a lower overpotential (307 mV), the results demonstrate that marine macroalgae extracts are a promising alternative for the green synthesis of efficient OER electrocatalysts. It should also be noted that some studies reported overpotentials at different current densities (1, 50, or 100 mA cm^–2^), and therefore direct quantitative comparisons should be interpreted with caution.

The electrochemical stability of the electrocatalysts was evaluated by subjecting them to 500 consecutive CV redox cycles (Figure S8). As observed, the CVs profiles recorded after 500 cycles exhibit only a slight shift relative to the initial curves, indicating good electrochemical stability of the materials. After 500 cycles, NiO-300 and NiO-700 exhibited modest increases in overpotential of 8 and 18 mV, respectively, whereas NiO-500 showed a slight decrease of 10 mV (Figure S9). These relatively small variations indicate that the electrodes largely retained their OER performance during accelerated electrochemical cycling. In Figure S10, the Raman spectra after OER of the working electrodes containing the NiO samples synthesized in this work can be observed. Compared to the NiO spectrum (Figure 4(b)), the spectral profile and band positions remain similar, differing mainly in intensity, which increases after OER. This behavior may be associated with the reorganization of active surface sites during prolonged electrochemical cycling, corroborating the LSV results after 500 CV redox cycles.^93^

EIS was employed to probe the physicochemical processes occurring at the electrode/electrolyte interface, providing deeper insights into the catalytic activity and reaction kinetics. The measurements were performed at three applied potentials (1.50, 1.55, and 1.60 V vs RHE) over a frequency range from 0.01 Hz to 10 kHz under oxygen evolution reaction (OER). The corresponding Nyquist and Bode plots are presented in Figure 9. The impedance spectra were fitted using the Randles equivalent circuit (*R*_s_(*R*_ct_ *Q*_CPE_)), assuming that the electrochemical process is governed by a single relaxation time constant (τ = RC). This circuit comprises the solution resistance (*R*_s_) in series with a parallel combination of the charge transfer resistance (*R*_ct_) and a constant phase element (CPE).^94,95^ The impedance of the CPE is defined as *Z*_CPE_ = [*Q*_CPE_(iω*^n^*)]^−1^, where *Q* represents the pseudocapacitive parameter and *n* (0 < *n* ≤ 1) is an empirical exponent associated with the deviation from ideal capacitive behavior, often reflected in the depression of the semicircle in the Nyquist plot. To obtain a more physically meaningful parameter, the CPE was converted into an effective capacitance using the relation *C* = *R*_ct_^(1 – *n*)/*n*^ *Q* ^1/*n*^, which was subsequently used to estimate the relaxation frequency corresponding to the apex of the semicircle.^96^ The fitted parameters derived from this model are summarized in Table 2.

**Table 2:** Values Obtained from the EIS Analyses

electrocatalyst	*R*_s_ (Ω)	*R*_ct_ (Ω)	*C* (mF)	*n*	*f*(Hz)
NiO-300					
1.50	0.7	32.7	79.9	0.8	0.06
1.55	0.6	4.2	26.0	0.7	1.45
1.60	0.6	1.5	12.3	0.7	8.76
NiO-500					
1.50	0.8	67.6	24.2	0.9	0.10
1.55	0.6	7.6	13.3	0.8	1.57
1.60	0.5	2.6	9.0	0.8	6.87
NiO-700					
1.50	0.8	77.8	19.5	0.9	0.11
1.55	0.50	8.5	9.3	0.8	2.01
1.60	0.50	2.9	6.0	0.8	9.09

**Figure 9:**
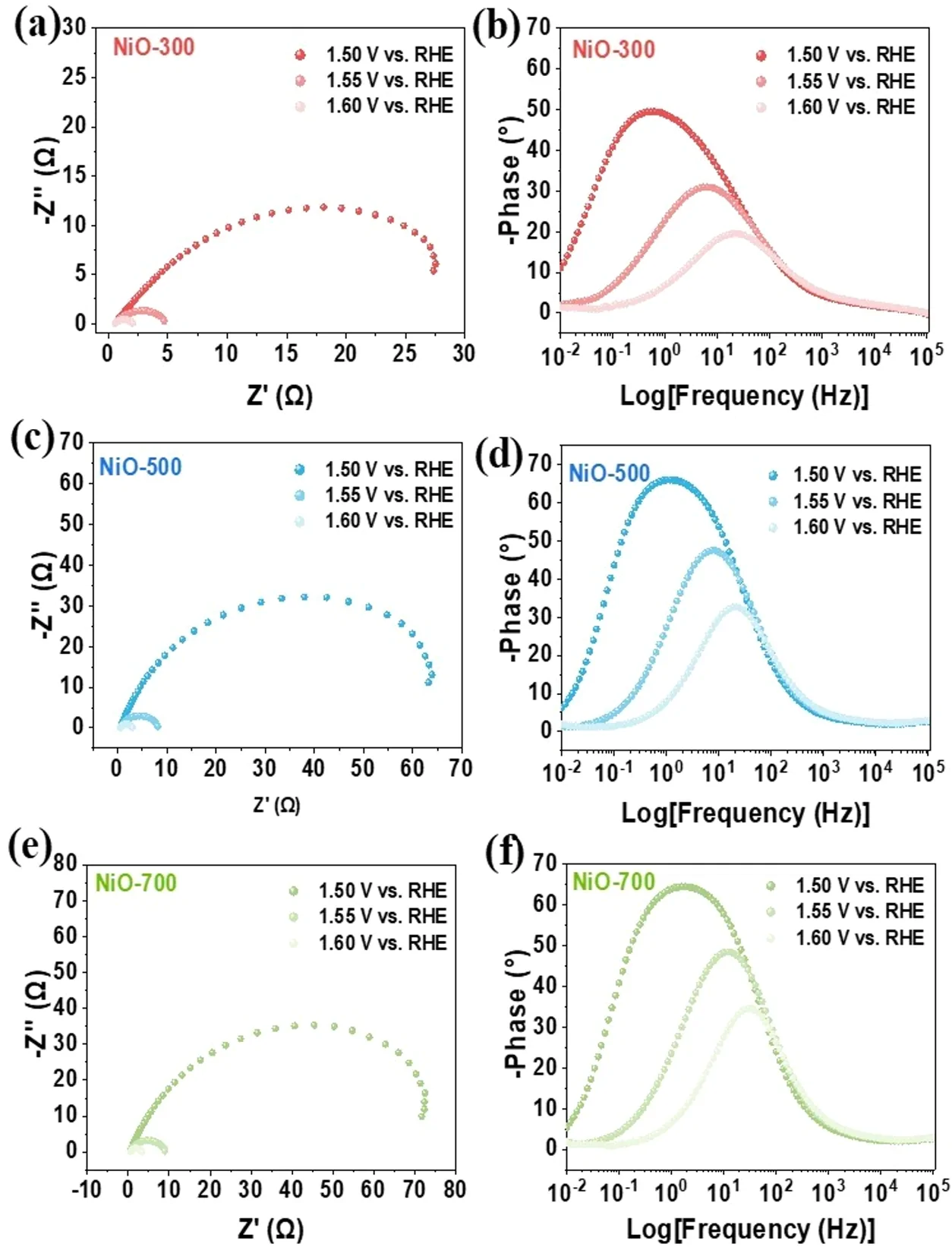
Nyquist (a, c, and e) and Bode (b, d, and f) plots for all NiO electrocatalysts.

As shown in Figures 9(a,c, and e), the diameter of the Nyquist semicircles progressively decreases with increasing applied potential (1.50, 1.55, and 1.60 V vs RHE), indicating a significant reduction in the charge transfer resistance (*R*_ct_) and, consequently, an acceleration of the OER kinetics.^97,98^ This trend is quantitatively supported by the fitted values (Table 2), where *R*_ct_ sharply decreases for all samples as the potential increases. For instance, NiO-300 exhibits a pronounced drop in *R*_ct_ from 32.7 Ω at 1.50 V to 1.5 Ω at 1.60 V, confirming a substantial improvement in interfacial charge transfer under more anodic conditions. Among the investigated electrocatalysts, NiO-300 consistently shows the lowest *R*_ct_ at all applied potentials, corroborating its superior catalytic performance toward the OER. This behavior is in excellent agreement with the LSV results at 10 mA cm^–2^, indicating that faster charge transfer kinetics play a critical role in its enhanced activity. In contrast, NiO-500 and NiO-700 display higher *R*_ct_ values, particularly at lower potentials, suggesting slower activation of catalytic sites. In addition to *R*_ct_, the evolution of the effective capacitance (*C*) and the CPE exponent (*n*) provide further insight into the electrode/electrolyte interface. The decrease in capacitance with increasing potential for all samples suggests a reduction in surface heterogeneity and/or changes in the adsorption behavior of reaction intermediates during the OER. The *n* values (0.7–0.9) indicate nonideal capacitive behavior, typically associated with surface roughness and structural heterogeneity, which are characteristic of nanostructured NiO materials.

The Bode plots (Figure 9(b,d, and f)) exhibit a single phase maximum, confirming that the electrochemical response is dominated by a single charge transfer process at the electrode/electrolyte interface.^99^ Notably, the characteristic frequency (*f*) shifts toward higher values with increasing potential (e.g., from 0.06 to 8.76 Hz for NiO-300), indicating a significant decrease in the relaxation time constant (τ = 1/2π*f*). This behavior reflects faster interfacial dynamics and improved charge transfer kinetics under OER operating conditions, in excellent agreement with the Nyquist analysis.

To further evaluate the electrochemical durability NiO-300 sample, chronopotentiometric (CP) measurements were performed at current densities of 10 and 100 mA cm^–2^ for 24 h (Figure 10(a)). In both cases, the potential remained stable throughout the entire test, with only negligible fluctuations and no evidence of progressive degradation. The NiO-300 catalyst required approximately 1.67 and 1.89 V vs RHE to sustain 10 and 100 mA cm^–2^, respectively, confirming its ability to operate under both moderate and industrially relevant current densities. The OER activity after the stability tests was further examined by LSV curves (Figure 10(b)). Following the CP measurements, the polarization curves remained very close to that of the pristine electrode, confirming that the catalyst retained most of its catalytic activity after continuous operation. Although a slight increase in overpotential was observed after the 100 mA cm^–2^ test, the overall electrocatalytic behavior remained well preserved, indicating that prolonged operation at elevated current densities induces only minor performance losses. These results demonstrate that the seaweed-derived NiO nanoparticles exhibit excellent electrochemical robustness under alkaline OER conditions.

**Figure 10:**
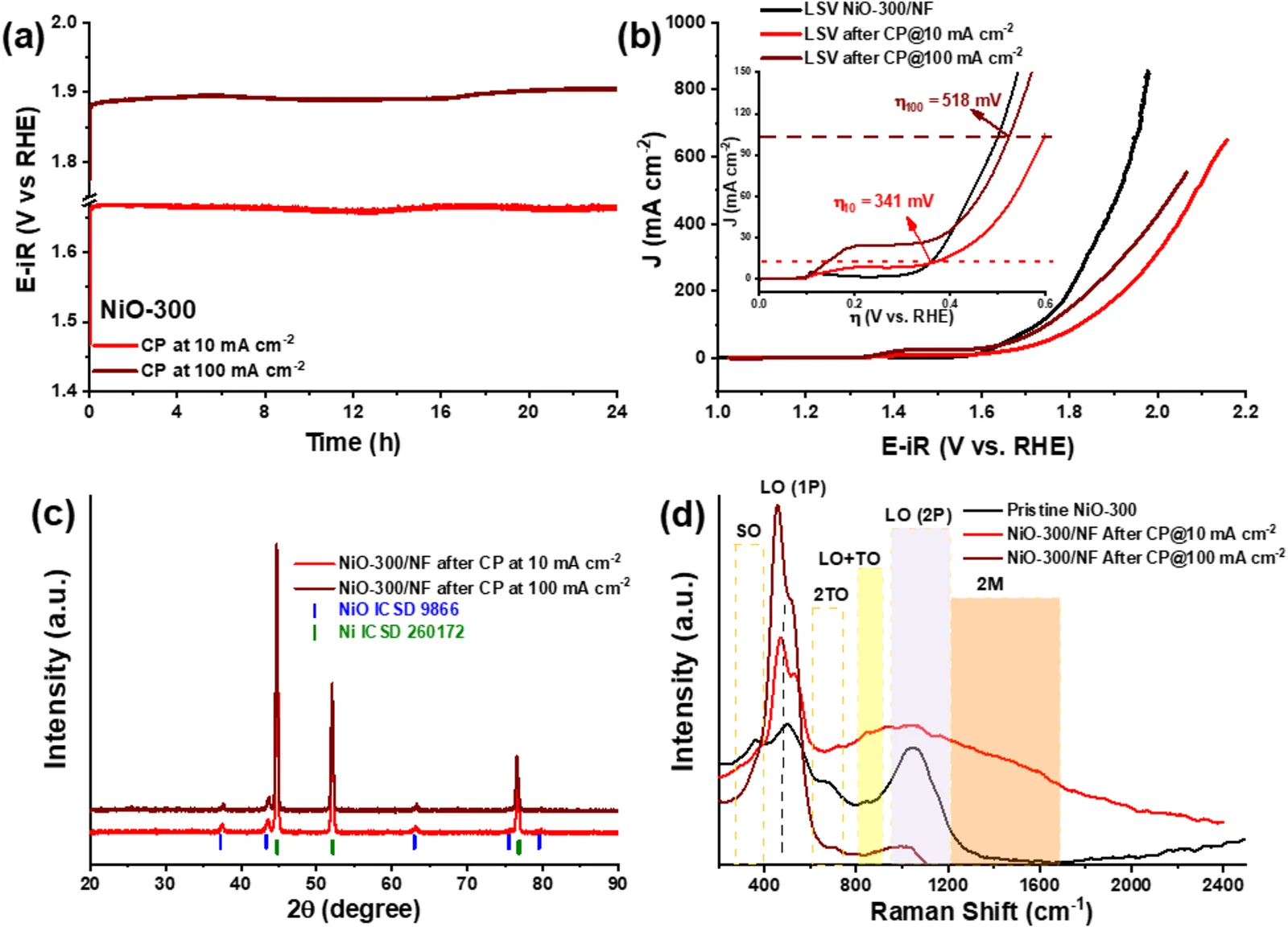
(a) Chronopotentiometric stability of the NiO-300/NF electrode at 10 and 100 mA cm^–2^ for 24 h in 1.0 M KOH. (b) LSV curves before and after the stability tests. Inset: corresponding overpotentials at 10 and 100 mA cm^–2^. (c) XRD patterns of the NiO-300/NF electrode after CP experiments. (d) Raman spectra of pristine NiO-300 and NiO-300/NF after CP experiments.

The structural integrity of the catalyst after medium-term electrolysis was verified by X-ray diffraction (Figure 10(c)). The diffraction patterns collected after CP measurements remain fully consistent with the cubic NiO phase (ICSD 9866), with no additional reflections associated with secondary phases. Furthermore, no appreciable peak shifts or significant broadening were observed after the stability tests, indicating that the crystalline was largely preserved during prolonged OER operation. These findings demonstrate the good electrochemical stability is accompanied by high structural stability. Additional insights into the surface evolution were obtained by Raman spectroscopy (Figure 10(d)). After the stability experiments, the Raman spectra preserve the characteristic vibrational features of pristine NiO-300 sample (Figure 4(b)), confirming that the oxide framework remains intact after prolonged electrolysis. At the same time, noticeable changes in the relative intensity and spectral profile of the phonon bands are observed, particularly after operation at 100 mA cm^–2^, suggesting that the catalyst surface undergoes structural reorganization during the OER. The sharper and more defined Raman features observed after electrolysis are also consistent with an increase in local structural ordering of the remaining NiO domains. Importantly, these modifications occur without evidence of bulk structural degradation, supporting that the electrocatalytic process predominantly involves surface reconstruction while preserving the crystalline NiO matrix.

## Conclusion

In summary, NiO nanoparticles were successfully synthesized through a sustainable route using an ethanolic extract of *Sargassum* sp. as a biogenic mediator. The calcination temperature strongly influenced the physicochemical properties of the materials, including crystallinity, particle growth, surface composition, and defect-related features. Among the samples investigated, NiO-300 exhibited the best OER performance, requiring an overpotential of 352 mV to reach 10 mA cm^–2^, whereas NiO-700 presented the lowest Tafel slope (69.4 mV dec^–1^). The superior activity of NiO-300, despite its lower estimated ECSA, together with its lower *R*_ct_ and the spectroscopic evidence provided by Raman, XPS, and EPR, indicates that the electrocatalytic performance is governed not only by the number of accessible active sites but also by the nature of the surface active sites and their associated electronic structure. Furthermore, chronopotentiometric measurements and post-OER XRD and Raman analyses demonstrated that the catalyst preserves its electrocatalytic activity and structural integrity after prolonged operation under alkaline conditions. Overall, this work demonstrates that seaweed-mediated synthesis is an effective and sustainable strategy for tailoring the electrocatalytic properties of NiO through controlled calcination. These findings broaden the application of marine biomass as a renewable platform for the preparation of efficient OER electrocatalysts.

## Supplementary Material



## Notes

Additional XRD, UV–Vis, TGA, XPS, SEM, electrochemical, and post-OER Raman data (PDF)

## References

[1] Khan I., Hou F., Le H. P. (2021). The Impact
of Natural Resources, Energy Consumption,
and Population Growth on Environmental Quality: Fresh Evidence from
the United States of America. Sci. Total Environ..

[2] Owusu P. A., Asumadu-Sarkodie S. (2016). A Review of
Renewable Energy Sources, Sustainability
Issues and Climate Change Mitigation. Cogent
Eng..

[3] Gielen D., Boshell F., Saygin D., Bazilian M. D., Wagner N., Gorini R. (2019). The Role of Renewable Energy in the
Global Energy Transformation. Energy Strategy
Rev..

[4] Sarker A. K., Azad A. K., Rasul M. G., Doppalapudi A. T. (2023). Prospect
of Green Hydrogen Generation from Hybrid Renewable Energy Sources:
A Review. Energies.

[5] Ajanovic A., Sayer M., Haas R. (2022). The Economics
and the Environmental
Benignity of Different Colors of Hydrogen. Int.
J. Hydrogen Energy.

[6] Fabbri E., Schmidt T. J. (2018). Oxygen Evolution
Reaction - The Enigma in Water Electrolysis. ACS Catal..

[7] Plevová M., Hnát J., Bouzek K. (2021). Electrocatalysts for
the Oxygen Evolution
Reaction in Alkaline and Neutral Media. A Comparative Review. J. Power Sources.

[8] Armaroli N., Balzani V. (2011). The Hydrogen Issue. ChemSusChem.

[9] Exner K. S. (2023). On the
Mechanistic Complexity of Oxygen Evolution: Potential-Dependent Switching
of the Mechanism at the Volcano Apex. Mater.
Horiz..

[10] Kumar S. S., Lim H. (2022). An Overview of Water Electrolysis Technologies for Green Hydrogen
Production. Energy Rep..

[11] Roger I., Shipman M. A., Symes M. D. (2017). Earth-Abundant
Catalysts for Electrochemical
and Photoelectrochemical Water Splitting. Nat.
Rev. Chem..

[12] Gou W., Zhang M., Zou Y., Zhou X., Qu Y. (2019). Iridium-Chromium
Oxide Nanowires as Highly Performed OER Catalysts in Acidic Media. ChemCatChem.

[13] Xiao M., Zhang Z., Tian Y., Miao Y., Wang J. (2017). Co–Fe–Se
Ultrathin Nanosheet-Fabricated Microspheres for Efficient Electrocatalysis
of Hydrogen Evolution. J. Appl. Electrochem..

[14] Shi H., Zha Q., Ni Y. (2022). Fe-Doped (Ni,Mn)­Co2O4 Nanorod Arrays on Ni Foam as
Highly Efficient Electrocatalyst for Oxygen Evolution Reaction in
Alkaline and Neutral Conditions with Superb Long-Term Stability. J. Alloys Compd..

[15] Samanta R., Shekhawat A., Sahu P., Barman S. (2024). Review and
Perspective
of Nickel and Its Derived Catalysts for Different Electrochemical
Synthesis Reactions in Alkaline Media for Hydrogen Production. Energy Fuel..

[16] Sivagami M., Asharani I. V. (2022). Phyto-Mediated Ni/NiO
NPs and Their Catalytic Applications-a
Short Review. Inorg. Chem. Commun..

[17] Vij V., Sultan S., Harzandi A. M., Meena A., Tiwari J. N., Lee W. G., Yoon T., Kim K. S. (2017). Nickel-Based Electrocatalysts
for Energy-Related Applications: Oxygen Reduction, Oxygen Evolution,
and Hydrogen Evolution Reactions. ACS Catal..

[18] Bonomo M. (2018). Synthesis
and Characterization of NiO Nanostructures: A Review. J. Nanopart. Res..

[19] Soltys L., Olkhovyy O., Tatarchuk T., Naushad M. (2021). Green Synthesis of
Metal and Metal Oxide Nanoparticles: Principles of Green Chemistry
and Raw Materials. Magnetochemistry.

[20] Mousa S. A., El-Sayed E.-S. R., Mohamed S. S., Abo El-Seoud M. A., Elmehlawy A. A., Abdou D. A. M. (2021). Novel Mycosynthesis
of Co3O4, CuO,
Fe3O4, NiO, and ZnO Nanoparticles by the Endophytic Aspergillus Terreus
and Evaluation of Their Antioxidant and Antimicrobial Activities. Appl. Microbiol. Biotechnol..

[21] Sagadevan S., Fatimah I., Lett J. A., Rahman M. Z., Leonard E., Oh W. C. (2023). Eco-Friendly Green
Approach of Nickel Oxide Nanoparticles for Biomedical
Applications. Open Chem..

[22] Singh P., Kim Y. J., Zhang D., Yang D. C. (2016). Biological Synthesis
of Nanoparticles from Plants and Microorganisms. Trends Biotechnol..

[23] Chaudhary R., Nawaz K., Khan A. K., Hano C., Abbasi B. H., Anjum S. (2020). An Overview of the Algae-mediated
Biosynthesis of Nanoparticles and
Their Biomedical Applications. Biomolecules.

[24] AlNadhari S., Al-Enazi N. M., Alshehrei F., Ameen F. (2021). A Review on Biogenic
Synthesis of Metal Nanoparticles Using Marine Algae and Its Applications. Environ. Res..

[25] Kumari, A. ; Pandey, A. A Review on Green Synthesis of Nickel Oxide Nanoparticles and Their Photocatalytic Activities Materials Today: Proceedings 2023 10.1016/j.matpr.2023.02.043.

[26] Chang X., Gao J., Han Y., Li L., Cao G., Zhang F., Sun S., She Y. (2026). Green Synthesis of
Nickel Oxide Nanoparticles for Enhanced
Oil Recovery: A Review. Energy Fuel..

[27] Garcia-Gonzalez F., Evans J. P. (2011). Fertilization Success and the Estimation of Genetic
Variance in Sperm Competitiveness. Evolution.

[28] Knoll A. H. (2014). Paleobiological
Perspectives on Early Eukaryotic Evolution. Cold Spring Harbor Perspect. Biol..

[29] Craveiro N., da Silva F., Nascimento M., Fechine J., Mangueira Y., Filho J. R. (2025). Chemical Compounds
from Seaweeds on the Tropical Coast
of Brazil. J. Braz. Chem. Soc..

[30] Craveiro N., Filho J. S. R. (2024). Macroalgae Traits
and Seasonality as Drivers of Polychaete
Assemblages on Macroalgae of Tropical Sandstone Reefs. Estuarine, Coastal Shelf Sci..

[31] Leandro A., Pereira L., Gonçalves A. M.
M. (2020). Diverse Applications
of Marine Macroalgae. Mar. Drugs.

[32] Dahoumane S. A., Mechouet M., Wijesekera K., Filipe C. D. M., Sicard C., Bazylinski D. A., Jeffryes C. (2017). Algae-Mediated Biosynthesis of Inorganic
Nanomaterials as a Promising Route in Nanobiotechnology-a Review. Green Chem..

[33] Mukherjee A., Sarkar D., Sasmal S. (2021). A Review of Green Synthesis of Metal
Nanoparticles Using Algae. Front. Microbiol..

[34] Akbar S., Tauseef I., Subhan F., Sultana N., Khan I., Ahmed U., Haleem K. S. (2020). An Overview
of the Plant-Mediated
Synthesis of Zinc Oxide Nanoparticles and Their Antimicrobial Potential. Inorg. Nano-Met. Chem..

[35] Si S., Mandal T. K. (2007). Tryptophan-Based
Peptides to Synthesize Gold and Silver
Nanoparticles: A Mechanistic and Kinetic Study. Chem. – Eur. J..

[36] Moavi J., Buazar F., Sayahi M. H. (2021). Algal Magnetic Nickel Oxide Nanocatalyst
in Accelerated Synthesis of Pyridopyrimidine Derivatives. Sci. Rep..

[37] Selvanathan V., Shahinuzzaman M., Selvanathan S., Sarkar D. K., Algethami N., Alkhammash H. I., Anuar F. H., Zainuddin Z., Aminuzzaman M., Abdullah H., Akhtaruzzaman M. (2021). Phytochemical-Assisted
Green Synthesis of Nickel Oxide Nanoparticles for Application as Electrocatalysts
in Oxygen Evolution Reaction. Catalysts.

[38] Zahra T., Ahmad K.s., Zequine C., Gupta R., Thomas A., Malik M. A., Iram S., ElBadry Y. A., El-Bahy Z. M. (2022). Electrochemical
Trapping of Meta-Stable NiO Consolidated ZnO/PdO by Biomimetic Provenance
for the Employment of Clean Energy Generation. Mater. Sci. Semicond. Process..

[39] da
Silva G. L., Hortêncio J., da S., Soares J. P. G., de S., Lourenço A., de A., Raimundo R. A., De Queiroz R. T., Macedo D. A., da Silva F. F. (2024). Phyto-Assisted Green
Synthesis of NiO Nanoparticles for OER Electrocatalysis. Int. J. Hydrogen Energy.

[40] Jaison J. P., Balasubramanian B., Gangwar J., Pappuswamy M., Meyyazhagan A., Kamyab H., Paari K. A., Liu W.-C., Taheri M. M., Joseph K. S. (2024). Bioactive Nanoparticles Derived from
Marine Brown Seaweeds and Their Biological Applications: A Review. Bioprocess Biosyst. Eng..

[41] Khalil A. T., Ovais M., Ullah I., Ali M., Shinwari Z. K., Hassan D., Maaza M. (2018). Sageretia Thea (Osbeck.)
Modulated
Biosynthesis of NiO Nanoparticles and Their in Vitro Pharmacognostic,
Antioxidant and Cytotoxic Potential. Artif.
Cells Nanomed. Biotechnol..

[42] Deka K., Nongbet R. D., Das K., Saikia P., Kaur S., Talukder A., Thakuria B. (2025). Understanding
the Mechanism Underlying
the Green Synthesis of Metallic Nanoparticles Using Plant Extract(s)
with Special Reference to Silver, Gold, Copper and Zinc Oxide Nanoparticles. Hybrid Adv..

[43] Heng M. H., Win Y. F., Cheah E. S. G., Chan Y. B., Rahman Md. K., Sultana S., Tey L.-H., Wong L. S., Djearamane S., Akhtaruzzaman M., Aminuzzaman M. (2024). Microwave-Assisted
Green Synthesis,
Characterization, and in Vitro Antibacterial Activity of NiO Nanoparticles
Obtained from Lemon Peel Extract. Green Process.
Synth..

[44] Drummer S., Madzimbamuto T., Chowdhury M. (2021). Green Synthesis
of Transition-Metal
Nanoparticles and Their Oxides: A Review. Materials.

[45] Medeiros S.
E. L., da Silva R. B., Gomes K. C., Silva V. D., Gonçalves J. A., Macedo D. A., Lourenço A. A., da Silva F. F., Azevedo S. (2023). Influence
of Particle Size on the Electrocatalytic Activity and Optical Properties
of NiO Nanoparticles. Mater. Sci. Eng., B.

[46] Bindu P., Thomas S. (2014). Estimation of Lattice
Strain in ZnO Nanoparticles:
X-Ray Peak Profile Analysis. J. Theor. Appl.
Phys..

[47] Xavier J., da Silva R. B., Araújo J. C. R., Iglesias C. A. M., Filho E. D. S., Fonseca J. L. C., Soares J. M., Souza P. B., Plá Cid C. C., Gamino M., de Medeiros S. N., Correa M. A., Bohn F. (2021). CoFe2O4@BiFeO3
Core/Shell Nanoparticles:
Synthesis, Characterization, and Fingerprints of the Spin Disorder. J. Alloys Compd..

[48] Nath D., Singh F., Das R. (2020). X-Ray Diffraction Analysis
by Williamson-Hall,
Halder-Wagner and Size-Strain Plot Methods of CdSe Nanoparticles-
a Comparative Study. Mater. Chem. Phys..

[49] Halder N. C., Wagner C. N. J. (1966). Separation of
Particle Size and Lattice Strain in Integral
Breadth Measurements. Acta Crystallogr..

[50] Rajith
Kumar C. R., Betageri V. S., Nagaraju G., Pujar G. H., Suma B. P., Latha M. S. (2020). Photocatalytic, Nitrite Sensing and
Antibacterial Studies of Facile Bio-Synthesized Nickel Oxide Nanoparticles. J. Sci.: Adv. Mater. Devices.

[51] Singh Y., Sodhi R. S., Singh P. P., Kaushal S. (2022). Biosynthesis of NiO
Nanoparticles Using Spirogyra Sp. Cell-Free Extract and Their Potential
Biological Applications. Mater. Adv..

[52] Roshid M.
H., Alam M. S., Amin K. A. I., Ferdousi F. K., Rahman M. H., Islam S. (2025). Green Synthesis
of Nickel Oxide Nanoparticles Using Lagerstroemia
Speciosa, Bombax Ceiba, and Piper Chaba Extracts and Evaluating Their
Potential Antioxidant, Antidiabetic and Antibacterial Properties. Heliyon.

[53] Kim J. H., Rho H., Kim J., Choi Y., Park J. (2012). Raman Spectroscopy
of ZnS Nanostructures. J. Raman Spectrosc..

[54] Cantoro M., Klekachev A. V., Nourbakhsh A., Sorée B., Heyns M. M., De Gendt S. (2011). Long-Wavelength,
Confined Optical
Phonons in InAs Nanowires Probed by Raman Spectroscopy. Eur. Phys. J. B.

[55] Sunny A., Balasubramanian K. (2020). Raman Spectral
Probe on Size-Dependent Surface Optical
Phonon Modes and Magnon Properties of NiO Nanoparticles. J. Phys. Chem. C.

[56] Duan W. J., Lu S. H., Wu Z. L., Wang Y. S. (2012). Size Effects on
Properties of NiO Nanoparticles Grown in Alkalisalts. J. Phys. Chem. C.

[57] Qiu J., Nguyen T. H., Kim S., Lee Y. J., Song M. T., Huang W. J., Chen X. B., Nguyen T. M. H., Yang I. S. (2022). Two-Dimensional
Correlation Spectroscopy Analysis of Raman Spectra of NiO Nanoparticles. Spectrochim. Acta, Part A.

[58] Maniammal K., Madhu G., Biju V. (2018). Nanostructured
Mesoporous NiO as
an Efficient Photocatalyst for Degradation of Methylene Blue: Structure,
Properties and Performance. Nano-Struct. Nano-Objects.

[59] Gandhi A. C., Pant J., Pandit S. D., Dalimbkar S. K., Chan T.-S., Cheng C.-L., Ma Y.-R., Wu S. Y. (2013). Short-Range
Magnon Excitation in NiO Nanoparticles. J. Phys.
Chem. C.

[60] Freitag A., Staemmler V., Cappus D., Ventrice C. A., Al Shamery K., Kuhlenbeck H., Freund H.-J. (1993). Electronic Surface State of NiO (100). Chem. Phys. Lett..

[61] Qi Y., Qi H., Li J., Lu C. (2008). Synthesis, Microstructures and UV–Vis
Absorption Properties of β-Ni­(OH)­2 Nanoplates and NiO Nanostructures. J. Cryst. Growth.

[62] Rahal H. T., Awad R., Abdel-Gaber A. M., Bakeer D. E.-S. (2017). Synthesis, Characterization,
and Magnetic Properties of Pure and EDTA-Capped NiO Nanosized Particles. J. Nanomater..

[63] Das R. K., Golder A. K. (2018). Use of Plant Based Analytes for the Synthesis of NiO
Nanoparticles in Catalyzing Electrochemical H2O2 Production. J. Electroanal. Chem..

[64] Bagus P. S., Nelin C. J., Brundle C. R., Crist B. V., Ilton E. S., Lahiri N., Rosso K. M. (2022). Main and
Satellite Features in the
Ni 2p XPS of NiO. Inorg. Chem..

[65] Grosvenor A. P., Biesinger M. C., Smart R., St C., McIntyre N. S. (2006). New Interpretations
of XPS Spectra of Nickel Metal and Oxides. Surf.
Sci..

[66] Biesinger M. C., Payne B. P., Grosvenor A. P., Lau L. W. M., Gerson A. R., Smart R. St. C. (2011). Resolving Surface Chemical States in XPS Analysis of
First Row Transition Metals, Oxides and Hydroxides: Cr, Mn, Fe, Co
and Ni. Appl. Surf. Sci..

[67] Biesinger M. C., Payne B. P., Lau L. W. M., Gerson A., Smart R. St. C. (2009). X-ray
Photoelectron Spectroscopic Chemical State Quantification of Mixed
Nickel Metal, Oxide and Hydroxide Systems. Surf.
Interface Anal..

[68] Sakamoto K., Hayashi F., Sato K., Hirano M., Ohtsu N. (2020). XPS Spectral
Analysis for a Multiple Oxide Comprising NiO, TiO2, and NiTiO3. Appl. Surf. Sci..

[69] Fu Z., Hu J., Hu W., Yang S., Luo Y. (2018). Quantitative
Analysis
of Ni2+/Ni3+ in Li­[NixMnyCoz]­O2 Cathode Materials: Non-Linear Least-Squares
Fitting of XPS Spectra. Appl. Surf. Sci..

[70] Sagui N. A., Ström P., Edvinsson T., Pehlivan İ. B. (2022). Nickel Site
Modification by High-Valence Doping: Effect of Tantalum Impurities
on the Alkaline Water Electro-Oxidation by NiO Probed by Operando
Raman Spectroscopy. ACS Catal..

[71] Sabat K. C. (2022). Production
of Nickel by Cold Hydrogen Plasma: Role of Active Oxygen. Plasma Chem. Plasma Process..

[72] Mihalcea C. G., Stefan M., Ghica C., Florea O. G., Stanoiu A., Simion C. E., Somacescu S., Ghica D. (2024). In-Depth Insight into
the Structural Properties of Nanoparticulate NiO for CO Sensing. Appl. Surf. Sci..

[73] Wagner, C. D. NIST X-Ray Photoelectron Spectroscopy (XPS) Database; Gaithersburg, MD 1990 10.6028/NIST.TN.1289.

[74] Barreca D., Bellucci A., Mastellone M., Trucchi D. M., Maccato C., Pierobon E., Gasparotto A., Rizzi G. A. (2025). Enhancing Oxygen
Evolution Performances of NiO-Based Electrocatalysts through Synergistic
Plasma Processing and Laser Treatment. Energy
Adv..

[75] Zhu J., Cannizzaro F., Liu L., Zhang H., Kosinov N., Filot I. A. W., Rabeah J., Brückner A., Hensen E. J. M. (2021). Ni–In Synergy in CO2 Hydrogenation to Methanol. ACS Catal..

[76] Chen X., Wang X., Fang D. (2020). A Review on C1s XPS-Spectra
for Some
Kinds of Carbon Materials. Fullerenes, Nanotubes
Carbon Nanostruct..

[77] Tahir M., Pan L., Idrees F., Zhang X., Wang L., Zou J. J., Wang Z. L. (2017). Electrocatalytic
Oxygen Evolution Reaction for Energy
Conversion and Storage: A Comprehensive Review. Nano Energy.

[78] Li G., Anderson L., Chen Y., Pan M., Chuang P.-Y. A. (2018). New
Insights into Evaluating Catalyst Activity and Stability for Oxygen
Evolution Reactions in Alkaline Media. Sustainable
Energy Fuel.

[79] Hales N., Schmidt T. J., Fabbri E. (2023). Reversible and Irreversible Transformations
of Ni-Based Electrocatalysts during the Oxygen Evolution Reaction. Curr. Opin. Electrochem..

[80] Jung S., McCrory C. C. L., Ferrer I. M., Peters J. C., Jaramillo T. F. (2016). Benchmarking
Nanoparticulate Metal Oxide Electrocatalysts for the Alkaline Water
Oxidation Reaction. J. Mater. Chem. A Mater..

[81] Zander J., Daumann F., Loukrakpam R., Roth C., Weber B., Marschall R. (2025). Correlations
of Calcination Temperature with the Catalytic
Properties of CuFe2O4 for the Synthesis of Green Fuels. Adv. Energy Sustainability Res..

[82] Zhou W., Lu X.-F., Chen J.-J., Zhou T., Liao P.-Q., Wu M., Li G.-R. (2018). Hierarchical
Porous Prism Arrays Composed of Hybrid
Ni–NiO–Carbon as Highly Efficient Electrocatalysts for
Overall Water Splitting. ACS Appl. Mater. Interfaces.

[83] Masood F., Hussain S., Arif M., Murtaza S., Imran M., Tahir M. B., Munawar K. S., Alkahtani H. M., Zen A. A., Shah S. A. A. (2025). Green Synthesis of Mangifera Indica
Mediated NiO and NiMn2O4 Nanomaterials for Electrocatalytic Water
Splitting. Int. J. Environ. Res..

[84] Sarkar D. K., Selvanathan V., Mottakin M., Hasan A. K. M., Islam M. A., Almohamadi H., Alharthi N. H., Akhtaruzzaman M. (2023). Phytochemical-Assisted
Green Synthesis of CuFeOx Nano-Rose Electrocatalysts for Oxygen Evolution
Reaction in Alkaline Media. RSC Adv..

[85] Zahra T., Ahmad K. S., Zequine C., Thomas A., Gupta R. K., Malik M. A., AA I. (2023). Electrocatalytic
Water Splitting
Studies on Zirconium Oxide Nanoparticles Synthesized by Biomimetic
Synthesis Route. Ionics.

[86] Zahra T., Ahmad K. S., Thomas A. G., Zequine C., Malik M. A., Gupta R. K. (2020). Organic Template-Based
ZnO Embedded Mn3O4 Nanoparticles:
Synthesis and Evaluation of Their Electrochemical Properties towards
Clean Energy Generation. RSC Adv..

[87] Azhar S., Ahmad K. S., Abrahams I., Ingsel T., Gupta R. K., El-marghany A. (2024). Synthesis
and Characterization Studies of Facile CuO:La2O3
Nanomaterial and Its Role as Electrode for Overall Energy Storage
and Energy Generation Applications. Chem. Pap..

[88] Azhar S., Ahmad K. S., Abrahams I., Lin W., Gupta R. K., Mazhar M., Ali D. (2021). Phyto-Inspired Cu/Bi
Oxide-Based
Nanocomposites: Synthesis, Characterization, and Energy Relevant Investigation. RSC Adv..

[89] Azhar S., Ahmad K. S., Abrahams I., Lin W., Gupta R. K., El-marghany A. (2023). Synthesis of Phyto-Mediated CuO–ZrO2
Nanocomposite
and Investigation of Their Role as Electrode Material for Supercapacitor
and Water Splitting Studies. J. Mater. Res..

[90] Alajmi M. F., Ahmed J., Hussain A., Ahamad T., Alhokbany N., Amir S., Ahmad T., Alshehri S. M. (2018). Green Synthesis
of Fe3O4 Nanoparticles Using Aqueous Extracts of Pandanus Odoratissimus
Leaves for Efficient Bifunctional Electro-Catalytic Activity. Appl. Nanosci..

[91] Zahra T., Ahmad K.s., Zequine C., Thomas A. G., Malik M. A., Gupta R. K., Ali D. (2021). Preparation
of Organo-Stabilized
Mn3O4 Nanostructures as an Electro-Catalyst for Clean Energy Generation. J. Electron. Mater..

[92] Sarkar D. K., Selvanathan V., Mottakin M., Islam M. A., Almohamadi H., Alharthi N. H., Akhtaruzzaman M. (2024). Phytochemicals
Assisted Green Synthesis
of Copper Oxide/Cobalt Oxide as Efficient Electrocatalyst for Oxygen
Evolution Reaction. Int. J. Hydrogen Energy.

[93] Dürr R. N., Maltoni P., Tian H., Jousselme B., Hammarström L., Edvinsson T. (2021). From NiMoO4
to γ-NiOOH: Detecting
the Active Catalyst Phase by Time Resolved in Situ and Operando Raman
Spectroscopy. ACS Nano.

[94] Chakthranont P., Kibsgaard J., Gallo A., Park J., Mitani M., Sokaras D., Kroll T., Sinclair R., Mogensen M. B., Jaramillo T. F. (2017). Effects
of Gold Substrates on the Intrinsic and Extrinsic
Activity of High-Loading Nickel-Based Oxyhydroxide Oxygen Evolution
Catalysts. ACS Catal..

[95] Lazanas A. Ch., Prodromidis M. I. (2023). Electrochemical
Impedance Spectroscopy–A Tutorial. ACS
Meas. Sci. Au.

[96] Raimundo R. A., Silva V. D., Medeiros E. S., Macedo D. A., Simões T. A., Gomes U. U., Morales M. A., Gomes R. M. (2020). Multifunctional
Solution Blow Spun NiFe–NiFe2O4 Composite Nanofibers: Structure,
Magnetic Properties and OER Activity. J. Phys.
Chem. Solids.

[97] Huang J., Han J., Wang R., Zhang Y., Wang X., Zhang X., Zhang Z., Zhang Y., Song B., Jin S. (2018). Improving
Electrocatalysts for Oxygen Evolution Using NixFe3xO4/Ni Hybrid Nanostructures
Formed by Solvothermal Synthesis. ACS Energy
Lett..

[98] Sun S., Jin X., Cong B., Zhou X., Hong W., Chen G. (2019). Construction
of Porous Nanoscale NiO/NiCo2O4 Heterostructure for Highly Enhanced
Electrocatalytic Oxygen Evolution Activity. J. Catal..

[99] Liang C., Liu L., Jia Z., Dai C., Xiong Y. (2015). Synergy of Nyquist
and Bode Electrochemical Impedance Spectroscopy Studies to Particle
Size Effect on the Electrochemical Properties of LiNi0.5Co0.2Mn0.3O2. Electrochim. Acta.

