# Supplementary material for: Sustainable Synthesis of Defect-Rich NiO Nanoparticles from Seaweed for Enhanced Oxygen Evolution
Source: ACS Omega. 2026 Aug 24;11(35):52943–58. doi: 10.1021/acsomega.6c05066 (PMC13563941; doi:10.1021/acsomega.6c05066)
Supplement: Supplementary file 1 [file ao6c05066_si_001.pdf]

Sustainable Synthesis of Defect-Rich NiO Nanoparticles from Seaweed for Enhanced Oxygen Evolution

Adiel Soares Ferreira,[a] Johnnys da Silva Hortêncio,[b] André Luiz Menezes de Oliveira,[c,d] Rodolfo B. da Silva,[e,f] Nykon Craveiro[g], José Souto Rosa-Filho[g*], Fausthon Fred da Silva[a,b,h,i]*.

[a] *Programa de Pós-Graduação em Química, Universidade Federal Rural de Pernambuco (UFRPE), 52171-900, Recife – PE Brazil.*

[b] *Programa de Pós-Graduação em Química, Universidade Federal da Paraíba (UFPB), 58.051-900, João Pessoa – PB, Brazil.*

[c] *Núcleo de Pesquisa e Extensão LACOM, Universidade Federal da Paraíba (UFPB), 58051-900, João Pessoa – PB, Brazil.*

[d] *Laboratório Institucional de Microscopia e Caracterização de Materiais, Depto. de Engenharia de Materiais, Universidade Federal do Rio Grande do Norte, 59078-970 Natal – RN, Brazil.*

[e] *Programa de Pós-Graduação em Ciência e Engenharia de Materiais - PPCEM, Universidade Federal da Paraíba (UFPB), 58.051-900, João Pessoa – PB, Brazil.*

[f] *Departamento de Física, Universidade do Estado do Rio Grande do Norte (UERN), 59610-090 Mossoró – RN, Brazil.*

[g] *Laboratório de Bentos (LaBen), Departamento de Oceanografia, Universidade Federal de Pernambuco, Av. Prof. Moraes Rêgo, 1235, 50670-901 Recife-PE, Brazil.*

[h] *Departamento de Química, Universidade Federal da Paraíba (UFPB), 58051-900, João Pessoa – PB, Brazil.*

[i] *Departamento de Química Fundamental, Universidade Federal de Pernambuco (UFPE), 50670-901, Recife-PE, Brazil.*

***Corresponding author:** souto.rosa@ufpe.br and fausthon@quimica.ufpb.br

SUPPLEMENTARY INFORMATION

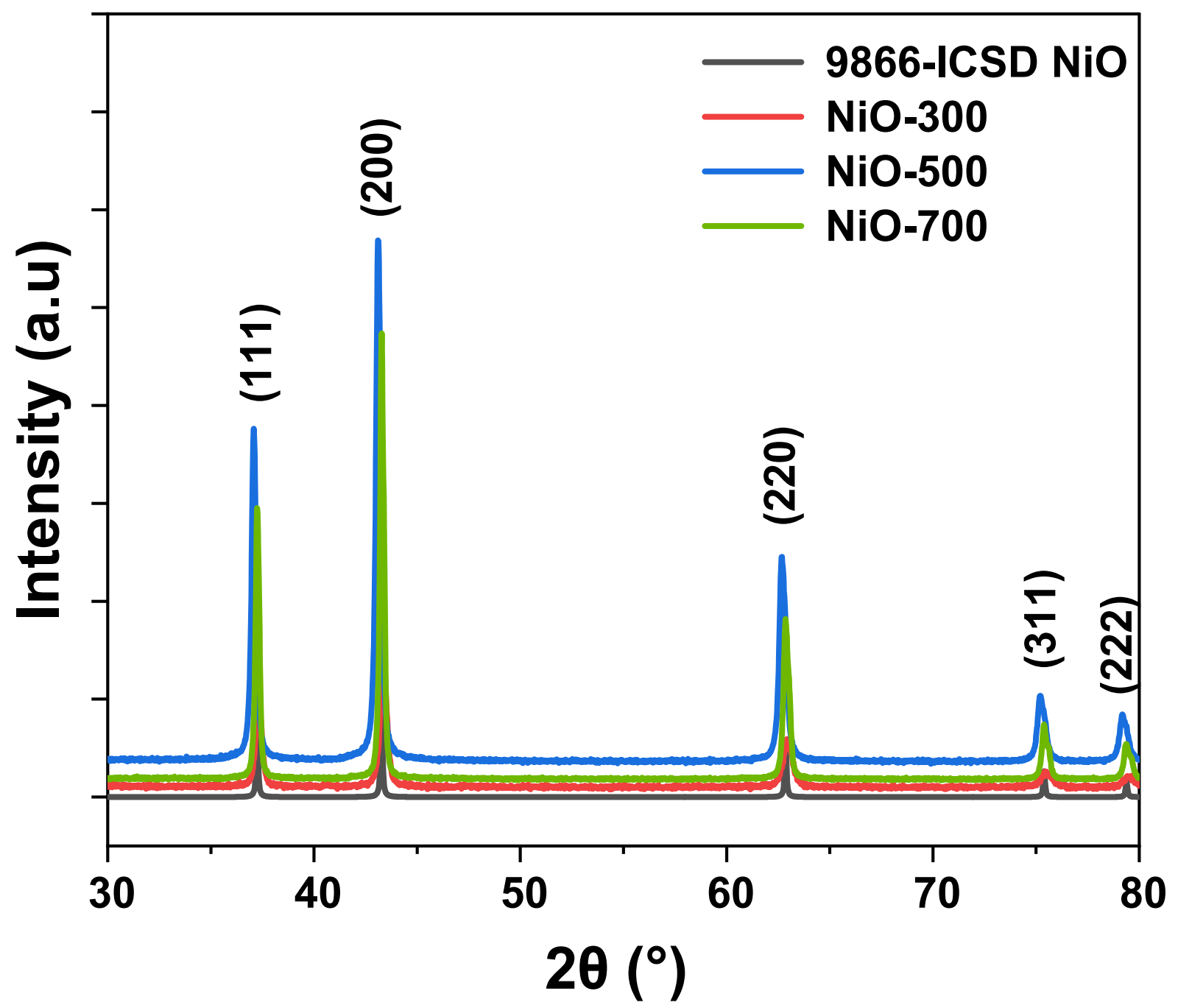

Figure S1. XRD patterns of NiO-300, NiO-500, and NiO-700 compared with the 9866-ICSD reference.

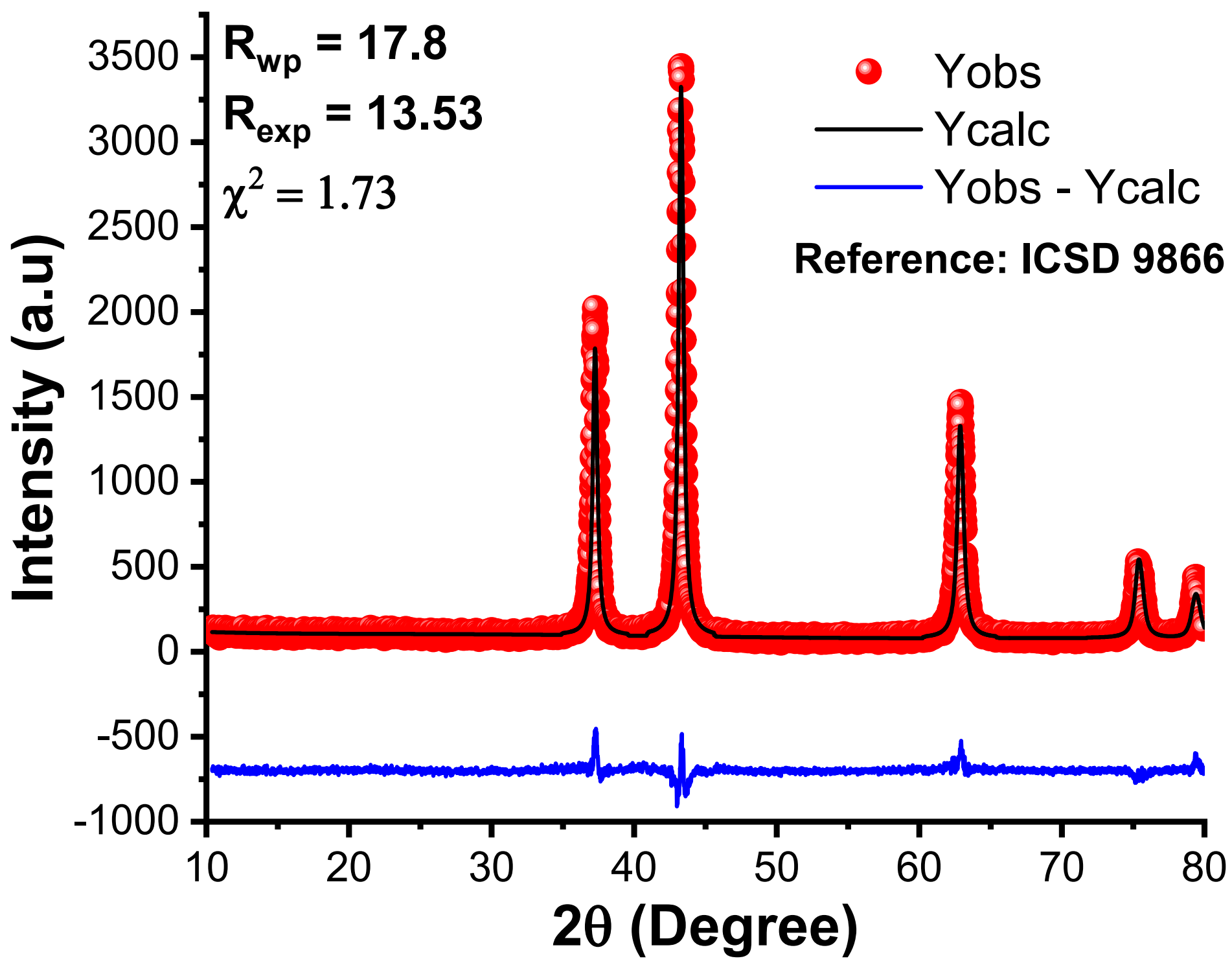

Figure S2. Rietveld refinement for the NiO(w) sample obtained using only water as solvent.

Table S1. Crystallite size (nm) and microstrain values of NiO samples calcined at different temperatures (300, 500, and 700 °C), calculated using the Scherrer method, Williamson–Hall (W–H), Size–Strain Plot (SSP), and Halder–Wagner (H–M) models.

Sample	Scherrer	W-H(size)	W-H(ε)	SSP-size	SSP-(ε)	H-M(size)	H-M(ε)
**NiO-300**	16.22	92.20	$1.57x10^{-3}$	48.42	$7.2x10^{-5}$	100.07	$2.0x10^{-5}$
**NiO-500**	23.22	35.83	$4.0x10^{-3}$	33.63	$3.8x10^{-6}$	155.62	$3.2x10^{-6}$
**NiO-700**	34.02	77.00	$1.8x10^{-3}$	50.56	$6.7x10^{-6}$	222.27	$4.4x10^{-6}$

Table S2. Lattice parameter (a) and refinement quality factor (χ^2) obtained from Rietveld refinement of the XRD patterns for the NiO samples calcined at different temperatures.

Sample	Lattice parameter	χ^2
**NiO-300**	a = 4.1780 Å	1.52
**NiO-500**	a = 4.1781 Å	1.67
**NiO-700**	a = 4.1795 Å	1.56

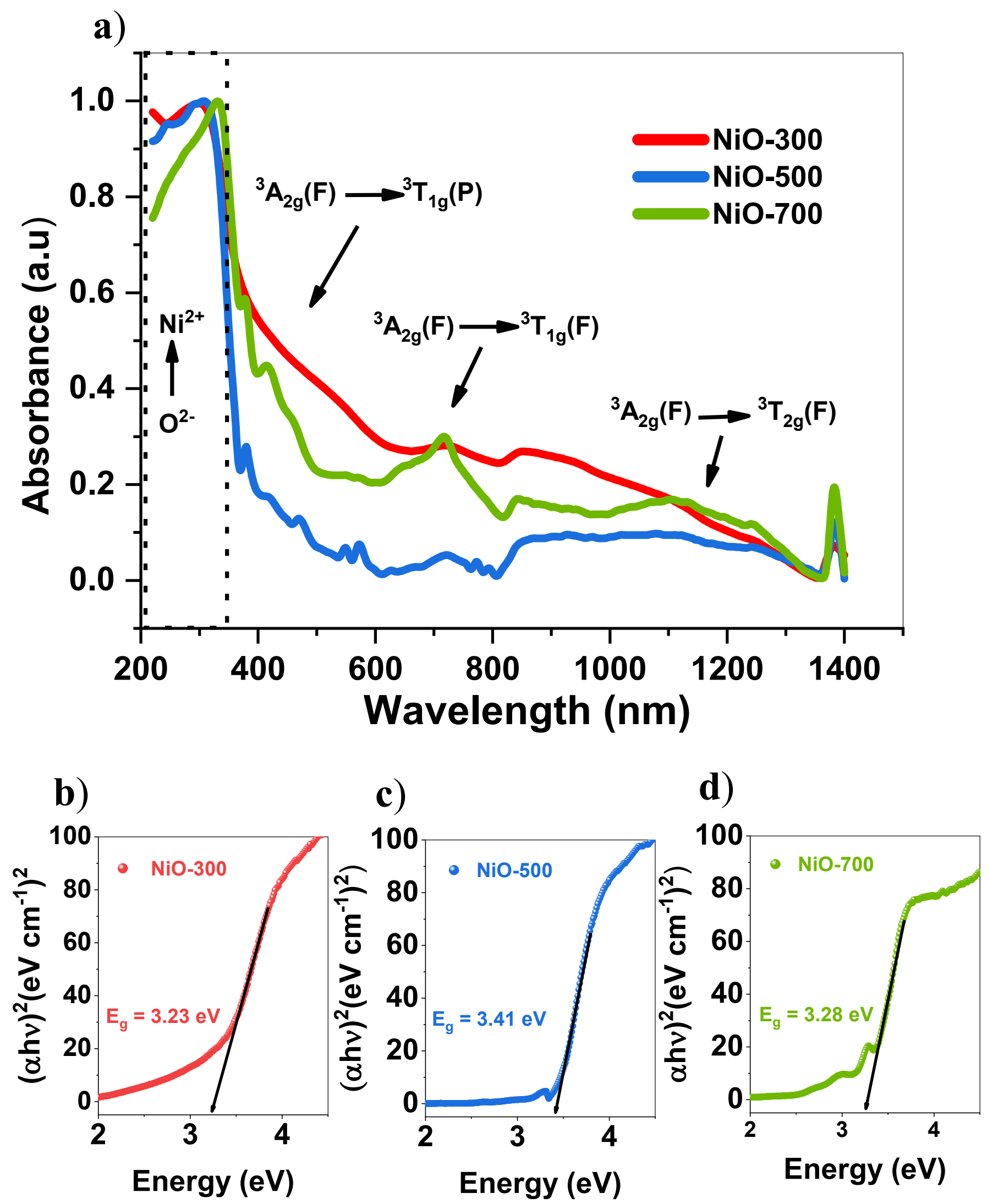

Figure S3. (a) UV-Vis solid state absorption spectra of the NiO samples, and (b-d) optical band gap determination of NiO-300, NiO-500, and NiO-700 using Tauc plots.

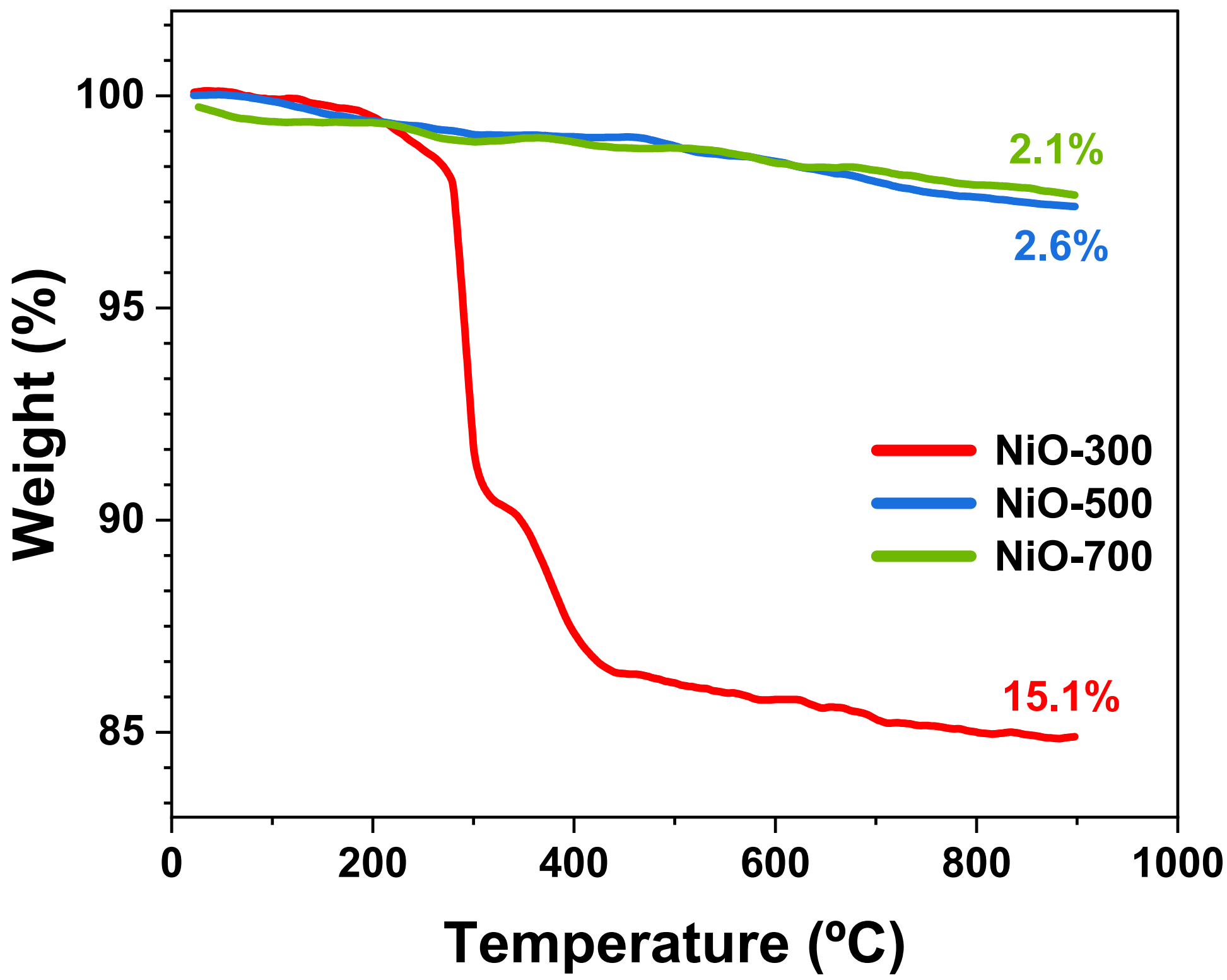

Figure S4. Thermogravimetric analysis (TGA).

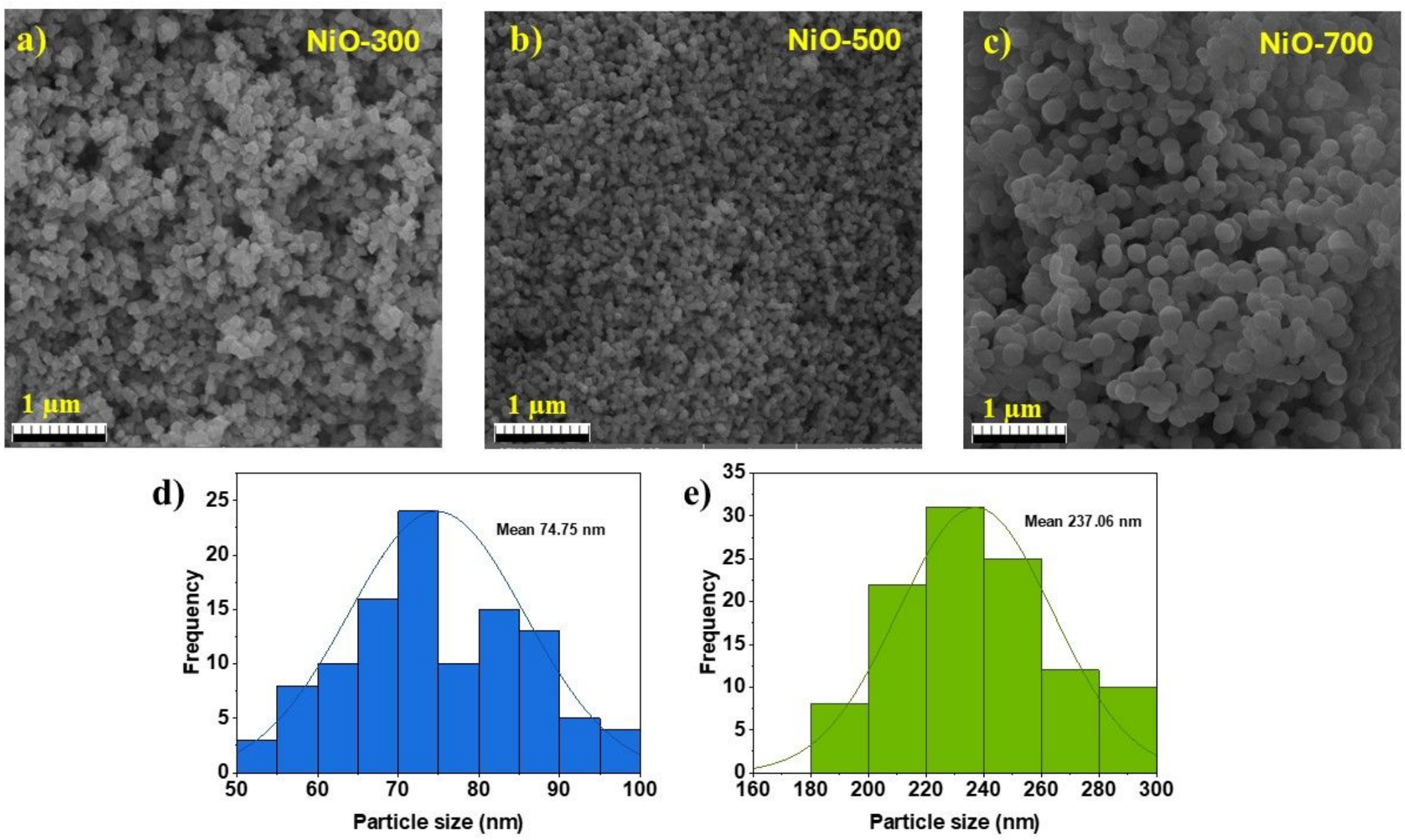

Figure S5. SEM images of nanoparticles: (a–c) morphology at different temperature calcination. (d–e) Particle size distribution histograms showing mean sizes of 74.75 nm and 237.06 nm for NiO-500 e NiO-700, respectively.

Table S3. Atomic percentages in the NiO samples obtained from the Survey XPS spectra.

	NiO-300		NiO-500		NiO-700	
	at%	StDev (%)	at%	StDev (%)	at%	StDev (%)
**Ni**	23.71	0.40	17.09	0.31	19.92	0.36
**O**	42.28	0.49	34.54	0.35	43.09	0.61
**C**	34.01	0.64	48.37	0.45	34.01	0.46

Table S4. Binding energy (eV) for the Ni $2p_{3/2}$ components of the NiO samples' spectra.

	NiO-300			NiO-500			NiO-700		
	Position (eV)	at%	ΔE (eV)	Position (eV)	at%	ΔE (eV)	Position (eV)	at%	ΔE (eV)
Ni^{2+}	853.75	24.11	1.72	853.71	25.25	1.71	853.78	24.49	1.68
Ni^{3+}	855.47	75.89		855.42	74.75		855.46	75.51	

Table S5. Binding energy (eV) for the O1s components of the NiO samples' spectra.

	NiO-300		NiO-500		NiO-700	
	Position	at%	Position	at%	Position	at%
**O_{struct} / Lattice oxygen (NiO)**	529.35	35.14	529.30	34.82	529.39	37.16
**O_{def} / OH**	530.82	58.67	530.93	55.08	530.88	54.42
**Adsorbed H_2O or O_2**	533.06	6.19	533.13	10.11	533.13	8.42

Table S6. Binding energy (eV) for the C1s components of the NiO samples' spectra.

	NiO-300	NiO-500	NiO-700
	Position	Position	Position
**C-C/ C=C**	284.76	284.62	284.71
**C-O**	286.01	285.78	285.97
**O-C=O**	288.67	288.38	288.37
**π-π***	292.82		

Table S7. Overpotential and Tafel slope values of NiO electrocatalysts obtained in this work compared with previously reported NiO-based electrocatalysts.

Electrocatalyst	η (mV) @10mA cm^{-2}	Tafel slope (mV dec^{-1})	Electrolyte	Ref.
**NiO-300**	**352**	**87.6**	**1.0 M KOH**	**This work**
**NiO-500**	**368**	**90.4**	**1.0 M KOH**	**This work**
**NiO-700**	**356**	**69.4**	**1.0 M KOH**	**This work**
Ni300H	307	60.35	1.0 M KOH	[1]
STNiO(111)	405	52.3	1.0 M KOH	[2]
Ni/NiO-NC	293	141	1.0 M KOH	[3]
NiO mesoporous	335	120	1.0 M KOH	[4]
$NiO_x(OH)_y$	380	40	1.0 M KOH	[5]
NiO_400 nm	345	48	1.0 M KOH	[6]
NiO	420	52	1.0 M KOH	[7]
Ni−NiO/C	295	52	1.0 M KOH	[8]
NiO nanorods	441	108	1.0 M KOH	[9]
Ni-NiO	370	90	1.0 M KOH	[10]
NiO + $NiCo_2O_4$	387	92.9	1.0 M KOH	[11]

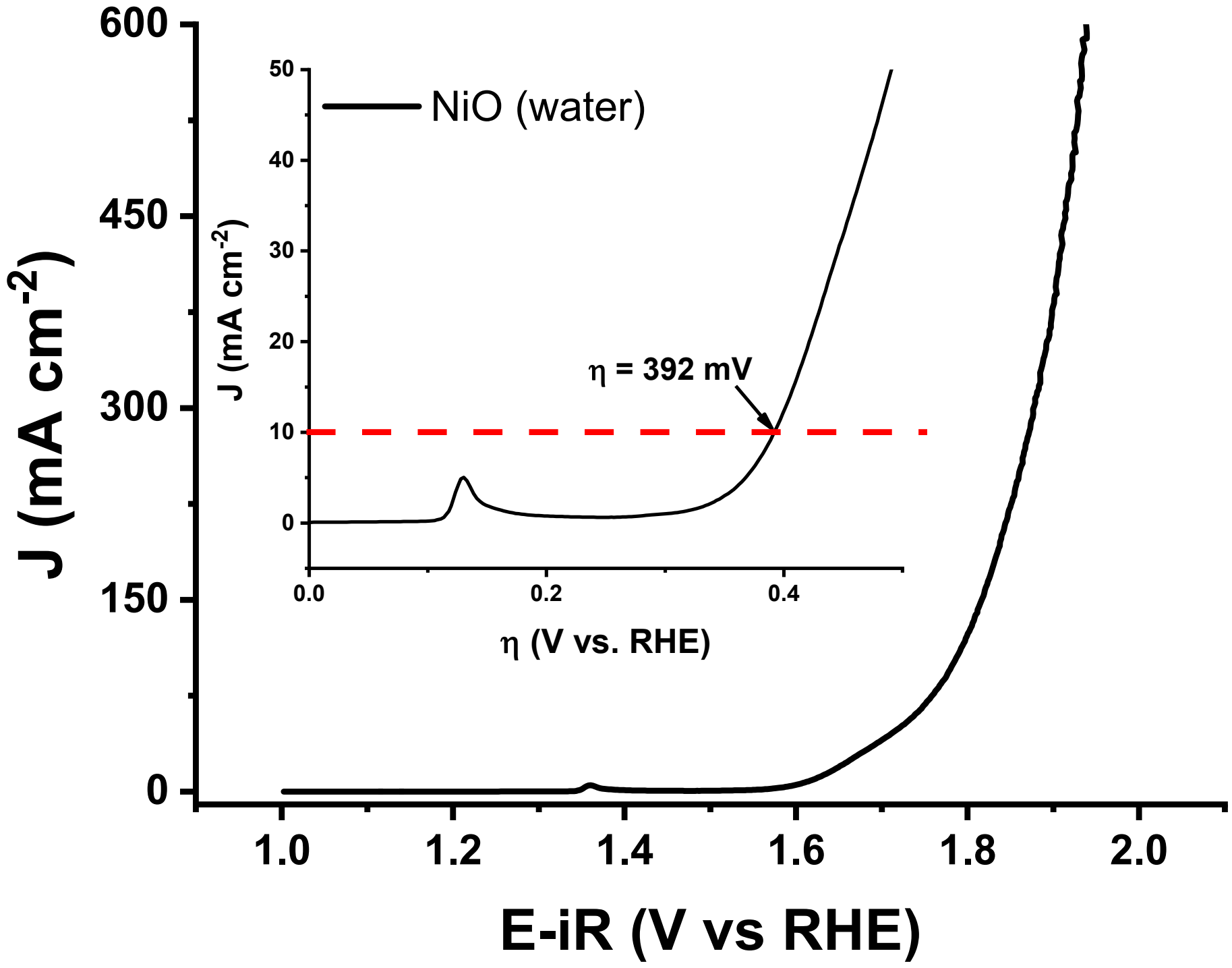

Figure S6. LSV of the NiO(w) sample, obtained in the absence of algae extract.

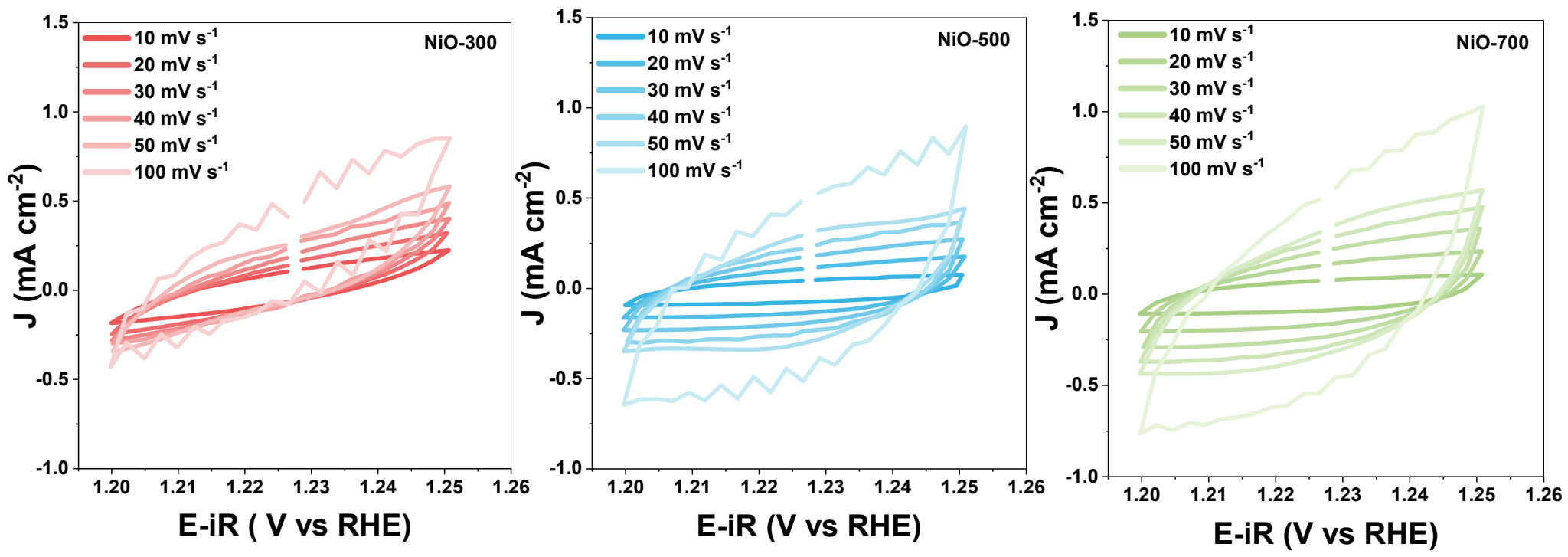

Figure S7. Cyclic voltammetry curves of NiO-300, NiO-500, and NiO-700 at different scan rates (10–100 mV s^{-1}).

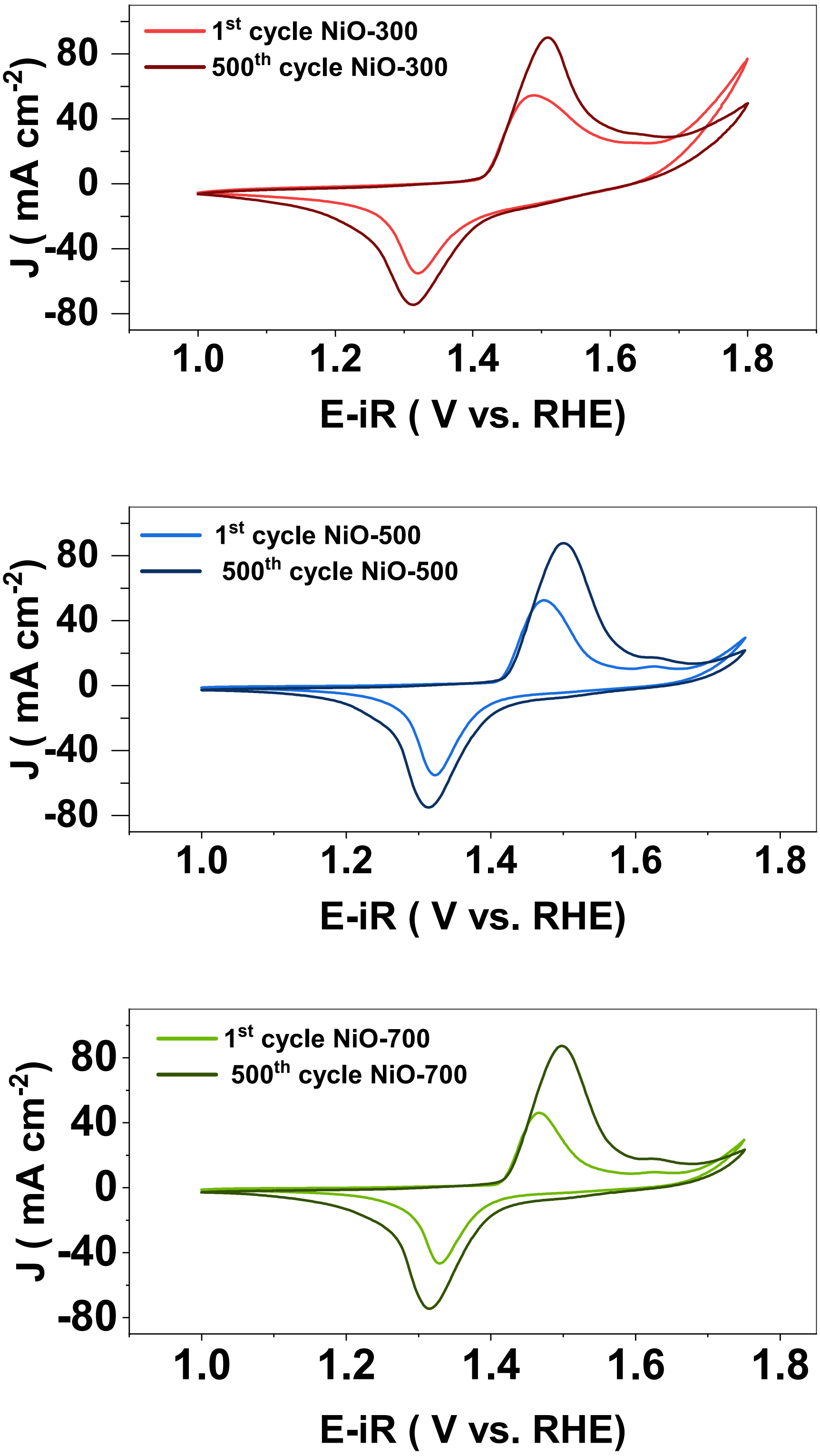

Figure S8. The first and 500th CV cycle for all electrocatalysts.

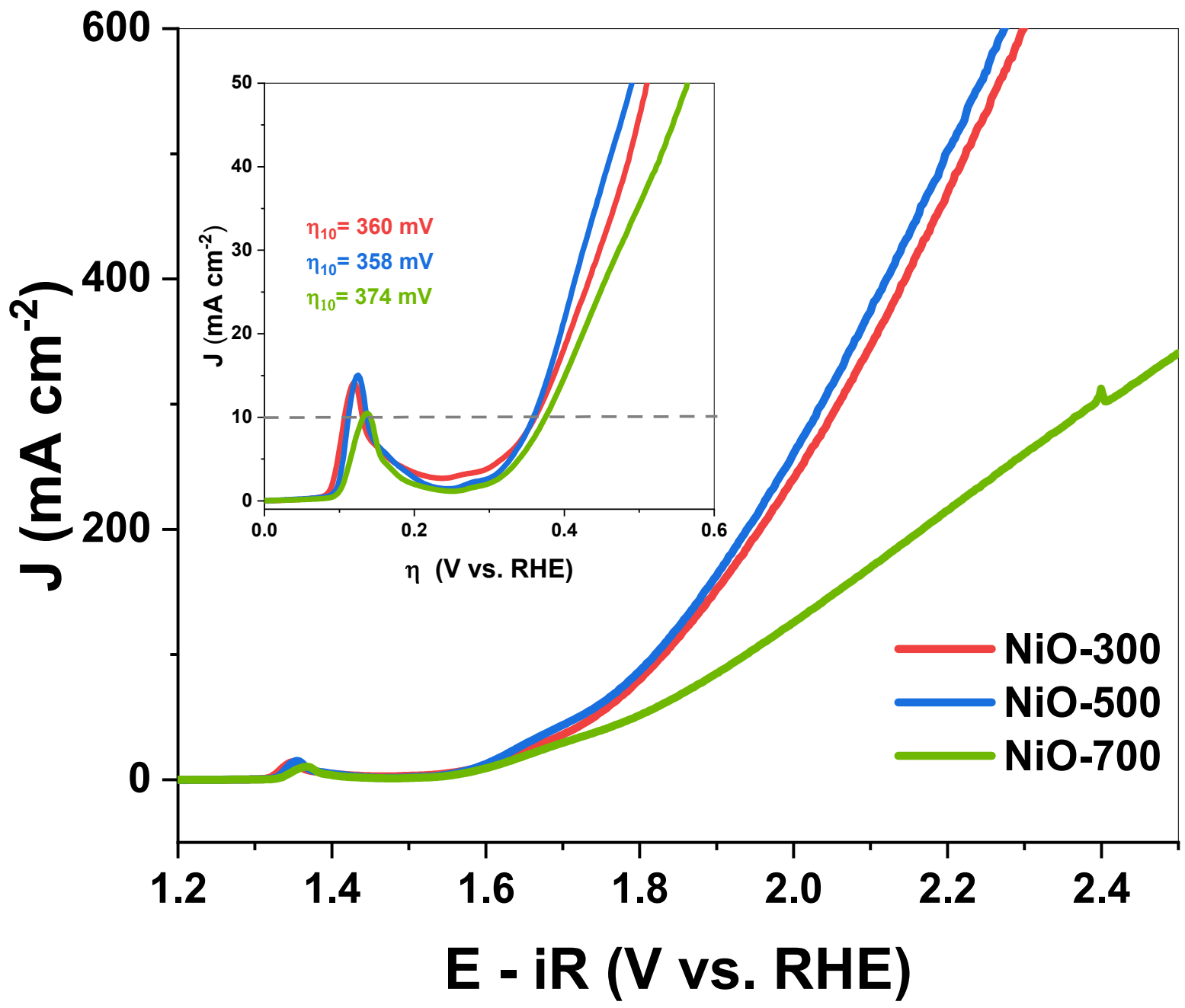

Figure S9. LSV curves for NiO-300, NiO-500, and NiO-700, after 500 CV cycles.

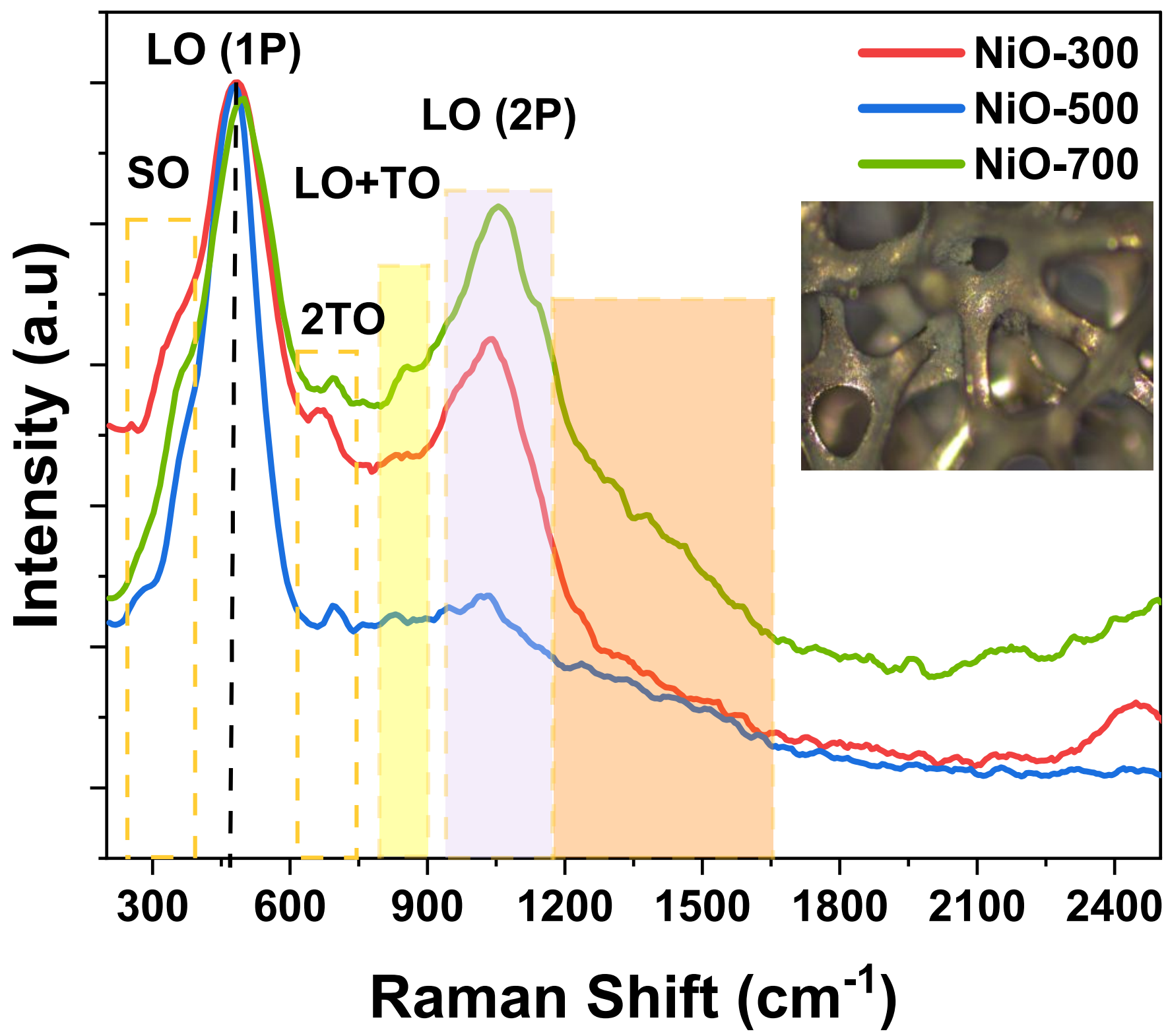

Figure S10. Raman vibrational spectra of electrode samples based on NiO after the oxygen evolution reaction (OER)

References

[1] da Silva GL, Hortêncio J da S, Soares JPG de S, Lourenço A de A, Raimundo RA, De Queiroz RT, et al. Phyto-assisted green synthesis of NiO nanoparticles for OER electrocatalysis. Int J Hydrogen Energy 2024;80:308–21. https://doi.org/10.1016/j.ijhydene.2024.07.160.

[2] Taffa DH, Brim E, Rücker KK, Hayes D, Lorenz J, Bisen O, et al. Influence of Annealing Temperature on the OER Activity of NiO(111) Nanosheets Prepared via Microwave and Solvothermal Synthesis Approaches. ACS Appl Mater Interfaces 2024;16:62142–54. https://doi.org/10.1021/acsami.4c14277.

[3] Liu Y, Mou G, Wang Y, He F, Dong N, Lin Y, et al. Nickel/Nickel Oxide-Nitrogen Self-Doped Carbon Nanosheets for Electrocatalytic Oxygen and Hydrogen Evolution Reactions. ACS Appl Nano Mater 2022;5:2953–61. https://doi.org/10.1021/acsanm.2c00222.

[4] Wu Z, Vagin M, Boyd R, Bakhit B, Greczynski G, Odén M, et al. Morphology effects on electrocatalysis of anodic water splitting on nickel (II) oxide. Microporous and Mesoporous Materials 2022;333. https://doi.org/10.1016/j.micromeso.2022.111734.

[5] Barreca D, Bellucci A, Mastellone M, Trucchi DM, Maccato C, Pierobon E, et al. Enhancing oxygen evolution performances of NiO-based electrocatalysts through synergistic plasma processing and laser treatment. Energy Advances 2025;4:1426–31. https://doi.org/10.1039/d5ya00273g.

[6] Cosentino S, Urso M, Torrisi G, Battiato S, Priolo F, Terrasi A, et al. High intrinsic activity of the oxygen evolution reaction in low-cost NiO nanowall electrocatalysts. Mater Adv 2020;1:1971–9. https://doi.org/10.1039/d0ma00467g.

[7] Kim SJ, Jo SG, Lee EB, Lee JW. Morphology-Controlled Nickel Oxide and Iron-Nickel Oxide for Electrochemical Oxygen Evolution Reaction. ACS Appl Energy Mater 2023;6:8360–7. https://doi.org/10.1021/acsaem.3c01046.

[8] Zhou W, Lu XF, Chen JJ, Zhou T, Liao PQ, Wu M, et al. Hierarchical Porous Prism Arrays Composed of Hybrid Ni-NiO-Carbon as Highly Efficient Electrocatalysts for Overall Water Splitting. ACS Appl Mater Interfaces 2018;10:38906–14. https://doi.org/10.1021/acsami.8b13542.

[9] Manjunath V, Bimli S, Singh D, Biswas R, Didwal PN, Haldar KK, et al. Porous nanorods by stacked NiO nanoparticulate exhibiting corn-like structure for sustainable environmental and energy applications. RSC Adv 2023;13:21962–70. https://doi.org/10.1039/d3ra03209d.

[10] Elizabeth I, Nair AK, Singh BP, Gopukumar S. Multifunctional Ni-NiO-CNT Composite as High Performing Free Standing Anode for Li Ion Batteries and Advanced Electro Catalyst for Oxygen Evolution Reaction. Electrochim Acta 2017;230:98–105. https://doi.org/10.1016/j.electacta.2017.01.189.

[11] Sun S, Jin X, Cong B, Zhou X, Hong W, Chen G. Construction of porous nanoscale NiO/NiCo2O4 heterostructure for highly enhanced electrocatalytic oxygen evolution activity. J Catal 2019;379:1–9. https://doi.org/10.1016/j.jcat.2019.09.010.
